# Fronto‐Parietal and Language Network Connectivity and Its Association With Gene Expression Profiles in Bipolar Disorder Before and After Treatment

**DOI:** 10.1111/cns.70236

**Published:** 2025-02-15

**Authors:** Leyi Zhang, Haohao Yan, Chunguo Zhang, Xiaoling Li, Jiaquan Liang, Chaohua Tang, Weibin Wu, Wen Deng, Guojun Xie, Wenbin Guo

**Affiliations:** ^1^ Department of Psychiatry, National Clinical Research Center for Mental Disorders, and National Center for Mental Disorders The Second Xiangya Hospital of Central South University Changsha Hunan China; ^2^ Department of Psychiatry The Third People's Hospital of Foshan Foshan Guangdong China

**Keywords:** Allen Human Brain Atlas, bipolar disorder, fronto‐parietal network, functional connectivity, functional magnetic resonance imaging, language network

## Abstract

**Background:**

The resting‐state functional connectivity (FC) patterns of the fronto‐parietal network (FPN) and language network (LN) underlying bipolar disorder (BD) are obscure. This study aimed to uncover abnormal FC patterns of FPN and LN underlying BD and their evolution following treatment.

**Methods:**

Imaging data at rest state and clinical variables were acquired from 82 patients with BD (with 43 finishing the follow‐up) and 88 healthy controls (HCs). Seed‐based FC analysis was performed, and correlations between FCs and clinical variables were investigated with whole‐brain multiple regression analyses. Furthermore, a neuroimaging–transcription spatial association analysis was conducted.

**Results:**

At baseline, BD patients presented elevated FPN‐LN and FPN–prefrontal gyrus FCs, and hyperconnectivity between the LN and bilateral thalamus, right angular gyrus (AG), and right cerebellum. Following 3 months of treatment intervention, there were decreased FCs between the FPN and left superior temporal gyrus (STG), left superior frontal gyrus (SFG), left insula, and bilateral middle temporal gyrus (MTG) (part of LN). Neuroimaging transcription analysis discovered genes correlated with FC alterations in BD.

**Conclusions:**

Aberrant FC patterns of FPN and LN might be involved in the neural pathogenetic and therapeutic mechanisms of BD. We also provided potential genetic pathways underlying these functional impairments in BD.

## Graphical abstract

Increased functional activity of the fronto‐parietal network (FPN) and language network (LN) might be involved in the pathogenetic mechanisms of bipolar disorder (BD). After treatment, baseline FCs were partially normalized, highlighting potential therapeutic mechanisms. Our neuroimaging–transcription analysis revealed genes and candidate pathophysiological processes associated with BD.

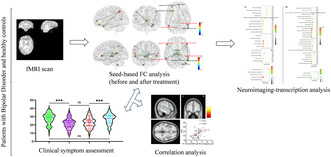

## 1 Introduction

Bipolar disorder (BD) is a chronic and recurrent neuropsychiatric condition characterized by the recurrent occurrence of depression, mania or hypomania, mixed episodes, and euthymia. It affects over 1% of the global population regardless of nation, ethnicity, or socioeconomic status [1]. Given that BD is a lifelong episodic illness, patients with BD may present functional and cognitive impairment, a decline in quality of life, and higher comorbidity and mortality [2, 3]. However, as of now, there are no valid biomarkers for BD. Finding new biomarkers for pathogenesis, diagnosis, and treatment response is therefore crucial.

Abundant functional magnetic resonance imaging (fMRI) studies have examined the pathophysiological and therapeutic mechanisms of BD and mainly found impairments within the prefrontal–limbic–subcortical circuit [4]. For instance, Ott et al. discovered hypoactivation in the dorsolateral prefrontal cortex (DLPFC) in remitted BD patients during working memory tasks. After 2 weeks of treatment, this pretreatment decreased activity was restored, serving as a potential biomarker for cognitive improvement [5]. However, the majority of BD‐related studies were task based, confounded by different task paradigms. The resting‐state fMRI (rs‐fMRI) technique offers a task‐free alternative, mitigating potential task confounders and facilitating repeated measurements. Using the graph theory metrics, Spielberg and his team found a decreased clustering coefficient of the right amygdala in the hypomanic group, along with an increase in the depressed group over an 8‐week course of lithium treatment [6]. These rs‐fMRI techniques possess the advantage of detecting spontaneous brain activity in BD patients, which accounts for 60%–80% of the brain's energy [7].

Furthermore, rs‐fMRI studies also provided insights into functional patterns of network connections, and several critical networks were consistently identified in BD during the rest state, including the default mode network (DMN) [8, 9], salience network (SN), and fronto‐parietal network (FPN) [10, 11]. Among them, FPN, as one of the core neurocognitive brain networks, is integral to working memory, sustained attention, and complex problem‐solving [12]. It is primarily composed of the DLPFC and posterior parietal cortex (PPC) [13]. Altered connections between FPN and several regions related to emotional processing might be conducive to distinguishing between BD and MDD [14]. The work of Rai demonstrated that aberrant DMN‐FPN FC underlined the deficiencies of BD in emotional processing, management, and regulation [10]. Another meta‐analysis suggested that FPN exhibited both hypoconnectivity and hyperconnectivity patterns between and within networks [15], so further research is required to clarify this contradiction. Abnormalities of the language network (LN), such as leftward language lateralization, have also been documented in BD patients [16, 17]. LN encompasses various parts of the Broadmann areas, including the inferior frontal gyrus (IFG), superior temporal gyrus (STG), and middle temporal gyrus (MTG). Disturbance in LN might account for the impulsivity, distractibility, and disorder of thoughts in BD [16]. Numerous studies have consistently clarified the aberrant IFG activations in BD patients and those at high risk [18, 19], and IFG‐based FC changes might contribute to emotional regulation and cognitive function [20, 21]. In young individuals with BD and at genetic high risk, Roberts and his colleagues reported dysconnectivity of the IFG between PFG (part of FPN) and STG, indicating that abnormal LN‐FPN connections and FC within the LN could be related to BD [22], but further studies were required to corroborate and expand these findings. Moreover, previous research on FC changes in BD was limited by a reliance on cross‐sectional designs, which failed to track the evolution of brain FCs following therapy. Longitudinal neuroimaging studies could offer significant insight into the therapeutic mechanisms.

Neuroimaging investigations have provided substantial evidence regarding potential brain functional and structural impairments for BD with limitations in understanding specific genetic mechanisms. BD has a high heritability (approximately 70%) [23] and genome‐wide association studies (GWAS) have revealed genetic variants related to imaging phenotypes [24]. Notably, neuroimaging–transcription association analysis holds the potential to uncover genetic mechanisms of disease‐related alterations in neuroimaging phenotypes based on whole‐brain gene expression data from the Allen Human Brain Atlas (AHBA) [25]. Using AHBA, a recent study identified genes related to homotopic FC alterations in schizophrenia patients [26]. In our previous study, we identified fractional ALFF‐related genes, with functional enrichment in biological pathways such as synaptic and ion transmission [27]. However, the correlations between FC changes and gene expression in BD are still unclear.

To our knowledge, the FC patterns of LN and FPN, and their trajectory following treatment in BD, remain equivocal. In the present study, we used eight seeds (bilateral lateral PFC (LPFC), PPC, IFG, and STG) of the FPN and LN to examine the FCs of these networks from the whole brain and conducted correlation analysis to explore the potential relationships between FCs and clinical variables. Furthermore, we aimed to observe FC changes after 3 months of therapeutic intervention. Finally, a cross‐sample spatial association analysis between the gene expression data from the AHBA and case–control FC changes was conducted in BD patients. Previous studies have reported both increased and decreased FCs within and between various FPN and LN regions. Given the prevalent presence of cognitive impairment in BD and the crucial role of FPN and LN in cognition, BD patients likely exhibit increased FCs of FPN and LN to compensate for impaired neural cognitive activity. Here, we hypothesized that BD patients would exhibit increased FCs within or between FPN, LN, and other regions involved in cognitive functions. These aberrant FCs at baseline might be partially normalized by treatment intervention. Significant correlations might be found between cognitive symptoms and these FCs.

## 2 Methods

### 2.1 Participants

We recruited a total of 82 BD patients from the Third People's Hospital in Foshan, China, for our research. Of these, 38 patients who received a 3‐month treatment completed the follow‐up. The inclusion criteria for BD patients were as follows: (1) confirmed by two psychiatrists according to the Statistical Manual of Mental Disorders, Fifth Edition (DSM‐5); (2) aged between 15 and 55 years; (3) right handed; and (4) at least 6 years of education. The exclusion criteria for BD patients were as follows: (1) presence of other psychiatric disorders, either past or present; (2) serious physical illness; (3) history of drug and alcohol abuse; (4) history of physiotherapy within the last 6 months; (5) pregnancy or breastfeeding; and (6) contraindications to MRI scanning.

Eighty‐eight HCs were sourced from the local community. If HCs showed signs of substance misuse, neurological illness, or psychosis, they were eliminated from enrollment. Any potential HCs with a personal or family history of psychiatric illness were excluded. Other exclusion criteria were the same as those for BD patients.

Following a comprehensive briefing regarding the project, all participants provided signed informed consent. The study, which was carried out in accordance with the Helsinki Declaration was approved by the Medical Ethics Committee of Foshan Third People's Hospital (FSSY‐LS201913).

### 2.2 Study Design

Before enrollment, all patients were undergoing pharmaceutical treatment. They were admitted due to conditions that had changed or worsened and were treated according to their conditions after being admitted. These patients underwent two MRI scans and clinical assessments, both at baseline and after 3 months of treatment. The HCs received one MRI scan and clinical assessment at baseline.

### 2.3 Assessments

The clinical symptoms of all the subjects were assessed using the Hamilton Depression Scale (HAMD‐24) [28], Hamilton Anxiety Scale (HAMA) [29], Bech‐Rafaelsen Mania Rating Scale (BRMS) [30], Social Disability Screening Schedule (SDSS) [31], Social Support Rating Scale (SSRS) [32], Simplified Coping Style Questionnaire (SCSQ) [33], Repeatable Battery for the Assessment of Neuropsychological Status (RBANS) [34], Stroop Color‐Word Test (SCWT), and Wisconsin Card Sorting Test (WCST) [35]. Their clinical indicators, including body mass index (BMI), thyroid stimulating hormone (TSH), free triiodothyronine (FT3), free thyroxine (FT4), triglycerides (TG), cholesterol (CHOL), high‐density lipoprotein (HDL), low‐density lipoprotein (LDL), fasting blood glucose (FBG), cortisol, uric acid, and heart rate (HR) were measured. Moreover, ERP (event‐related potentials) and EEM (exploratory eye movement) were conducted to understand their cognitive processes and visual perception and attention. Details on the measurement of ERP are shown in Appendix S1.

### 2.4 Imaging Data Acquisition and Preprocessing

The rs‐fMRI data were collected using a 3.0 T GE scanner with a gradient echo‐planar imaging sequence: repetition time/echo time = 2000/30 ms, number of slices=36, matrix size=64 × 64, flip angle = 90°, field of view=220 × 220 mm, slice thickness= 4 mm, slice gap = 0 mm, voxel size= 3.75 × 3.75 × 4.00 mm, and 250 volumes. T1‐weighted images were obtained with a 3D fast spoiled gradient‐echo sequence with the following parameters: repetition time/echo time= 8.58/3.30 ms, number of slices=172, matrix size= 256 × 256, flip angle= 9°, field of view= 256 × 256 mm, slice thickness= 1 mm, slice gap =0 mm, and voxel size= 1 × 1 × 1 mm. All the participants were instructed to stay motionless, keep their eyes closed, and maintain wakefulness. The rs‐fMRI data were preprocessed in MATLABR2018b (http://www.mathworks.com) with the SPM12 and RESTplus software. Next, we applied the following preprocessing steps: (1) removal of the initial 10 time points; (2) slice timing; (3) realignment; (4) registration of BOLD images to individual T1‐weighted images and normalization to MNI space; (5) spatial smoothing with a 4 mm full‐width half‐maximum kernel; (6) linear detrending; (7) regression of covariates; and (8) bandpass filtering. See Appendix S1 for more details on imaging preprocessing.

### 2.5 ROIs Selection and FC Analysis

As previous studies suggested [21, 36, 37], eight seeds involved in the FPN [ROI1–ROI4, LPFC (−43, 33, 28) and (41, 38, 30), and PPC (−46, −58, 49) and (52, −52, 45)], and LN [ROI5‐ROI8, IFG (−51, 26, 2) and (1, 28, 54), and STG (57, −47, 15) and (59, −42, 13)] were chosen as ROIs (Table S1), provided by the CONN functional connectivity (FC) toolbox [38]. Each ROI was defined as a sphere with a radius of 6 mm. By averaging the time series of the voxels within the ROI, the time series of each seed was computed. We produced correlation maps by computing the Pearson correlation coefficients between each ROI's time course and those of other voxels throughout the entire brain. Fisher's r‐to‐z transformation was then used to enhance the normality of their distribution.

### 2.6 Statistical Analysis

The normality test for continuous data was performed using histograms and the Shapiro–Wilk test. Differences in demographic and clinical characteristics between BD patients and HCs were examined with the Mann–Whitney *U* test or two‐sample *t*‐tests in SPSS 25.0 software, if applicable. Categorical data were evaluated using chi‐square tests. The clinical features of BD patients at baseline and those who completed the follow‐up were compared using paired *t*‐tests or Wilcoxon signed‐rank tests.

Two sample *t*‐tests were conducted to compare the FCs of the FPN and LN between HCs and BD patients (both the whole BD group and manic BD, respectively), with age, gender, educational level, and head movement (mean framewise displacement) as covariates. To detect FC changes following therapy, paired *t*‐tests were used on BD at baseline with those after treatment. The Gaussian random field (GRF) theory was employed to correct multiple comparisons, with a cluster significance of *p* < 0.05 and voxel significance of *p* < 0.001. Correlations between FCs and clinical variables were investigated with whole‐brain multiple regression analyses on the SPM toolbox, with age, gender, and educational level as covariates. The familywise error (FWE) correction (*p* < 0.05) was applied to correct the significance level of multiple regression for multiple comparisons.

### 2.7 Neuroimaging–Transcription Association Analysis

**FIGURE 1:**
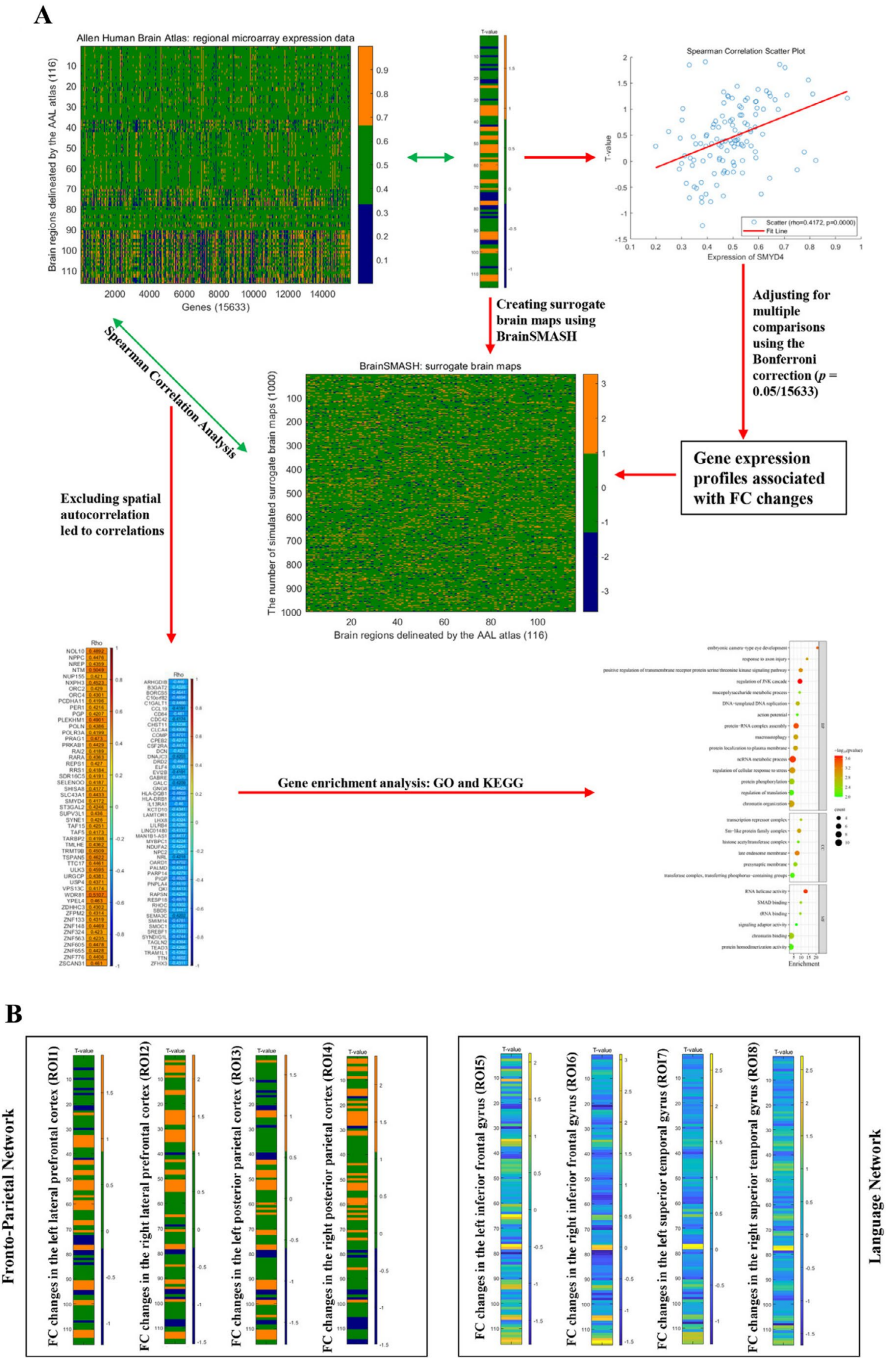
(A) A flowchart that outlines the transcriptome–neuroimaging spatial correlation analyses and enrichment analyses. First, we preprocessed the gene data from the Allen Human Brain Atlas database using the Abagen toolbox, obtaining a 15,633 × 116 gene expression matrix. Spearman correlation analysis was then performed to calculate the correlation between changes in FC and gene expression, with the significance level adjusted using Bonferroni correction. Next, the BrainSMASH toolbox was applied to exclude the potential impact of spatial autocorrelation on the correlation between changes in FC and gene expression. Finally, genes associated with FC changes were subjected to enrichment analysis using the Metascape online toolbox. (B) Changes in FC of the ROIs from the fronto‐parietal and language networks. These FC maps were used to correlate with the gene expression data from the Allen Human Brain Atlas database. AAL Atlas, Automated Anatomical Labeling Atlas; FC, functional connectivity; GO, Gene Ontology; KEGG, Kyoto Encyclopedia of Genes and Genomes; ROI, region of interest.

A flowchart that outlines the transcriptome–neuroimaging spatial correlation analyses is shown in Figure 1A. The gene expression data were sourced from the AHBA database. We followed a standard pipeline to process the brain transcriptomic data [39]. For details, please see Appendix S1. Using the Abagen toolbox (version 0.1.1; https://github.com/rmarkello/abagen), a 15,633 × 116 (genes × regions) gene expression matrix was obtained. Based on the *T*‐maps of the case–control FC alterations between different ROIs and the whole brain at baseline (Figure 1B), gene‐wise cross‐sample associations between FC differences and gene expression were analyzed by Spearman correlation. The significance level was adjusted by the Bonferroni correction (*p* < 0.05/15633) for multiple comparisons.

Considering the impact of autocorrelation in neuroimaging maps, we applied the BrainSMASH toolbox [40] to generate 1000 surrogate brain maps for each *T*‐map. The same Spearman correlation analysis was repeated using these surrogate maps. Genes whose expression patterns exhibited a correlation with 50 of 1000 surrogate brain maps were excluded from further analysis (*p* < 0.05). Subsequently, FC alteration‐related genes were subjected to Gene Ontology (GO) and Kyoto Encyclopedia of Genes and Genomes (KEGG) enrichment analysis with Metascape (https://metascape.org/).

## 3 Results

### 3.1 Demographic and Clinical Characteristics

Five BD patients at baseline, five BD patients who finished the follow‐up, and five HCs were excluded during the processing of the imaging data due to excessive head movement (displacement greater than 2 mm or angular motion exceeding 2° in the *x*, *y*, or *z* axes). As a result, a total of 77 BD patients (38 BD patients finished the follow‐up) and 83 HCs were included in the final analysis. As shown in Table 1, the BD and HC groups were comparable in terms of age, gender, and years of education (*p* > 0.05). Their psychological and cognitive statuses are displayed in Tables S2 and S3.

**TABLE 1:** Demographic and clinical characteristics.

Variables	Patients (mean ± SD, *n* = 77)	Controls (mean ± SD, *n* = 83)	*p*
Age (years)	30.44 ± 8.88	34.84 ± 11.93	0.074^a^
Gender (male/female)	28/49	33/50	0.659^b^
Years of education (years)	12.99 ± 3.06	13.24 ± 3.67	0.096^a^
Illness duration (months)	88.35 ± 71.65	/	/
Manic/depressive/hypomanic/mixed episodes	59/18/0/0	/	/
Follow‐up time points (months)	3	/	/
BMI (kg/m^2^)	23.62 ± 4.33	22.83 ± 3.70	0.304^a^
TSH (mIU/L)	1.69 ± 1.16	2.47 ± 2.22	< 0.001^a^
FT3 (pmol/L)	4.67 ± 0.78	4.80 ± 0.59	0.269^c^
FT4 (pmol/L)	15.54 ± 3.54	15.61 ± 3.03	0.766^a^
TG (mmol/L)	1.19 ± 0.75	1.28 ± 0.77	0.199^a^
CHOL (mmol/L)	4.31 ± 1.00	4.75 ± 0.79	< 0.001^a^
HDL (mmol/L)	1.19 ± 0.31	1.25 ± 0.31	0.193^a^
LDL (mmol/L)	2.51 ± 0.86	2.62 ± 0.64	0.056^a^
FBG (mmol/L)	6.21 ± 1.41	5.61 ± 0.80	0.024^a^
Cortisol (nmol/L)	355.11 ± 142.65	323.60 ± 114.15	0.136^c^
Uric acid (μmol/L)	361.45 ± 116.45	356.46 ± 89.57	0.763^c^
HR (times/min)	83.47 ± 17.21	68.04 ± 10.22	< 0.001^a^
QRS complex (ms)	90.68 ± 10.12	96.29 ± 10.63	0.001^a^
PR interval (ms)	144.07 ± 20.97	153.95 ± 17.95	0.002^c^
QTc (ms)	361.33 ± 28.73	392.43 ± 25.94	< 0.001^c^

Abbreviations: BMI, body mass index; CHOL, cholesterol; FBG, fasting blood glucose; FT3, free triiodothyronine; FT4, free thyroxine; HDL, high‐density lipoprotein; HR, heart rate; LDL, low‐density lipoprotein; SD, standard deviation; TG, triglyceride; TSH, thyroid stimulating hormone.

^a^ The *p*‐values were obtained by a Mann–Whitney *U* test.

^b^ The *p*‐value for sex distribution was obtained by a Chi‐Square test.

^c^ The *p*‐values were obtained by a two‐sample *t* test.

### 3.2 The Treatment Outcome

The clinical indicator and assessment changes of 38 follow‐up BD patients are shown in Tables S4–S6. After 3 months of treatment, the scores of BRMS, HAMD, HAMA (*p* < 0.001), SDSS (*p* = 0.005), and RBANS (*p* = 0.001), and the subscales of WCST, such as perseverative response (*p* = 0.008), changed significantly. Figures S1–S8 show the differences in clinical variables across groups.

### 3.3 FC Analyses Between BD Patients and HCs at Baseline

**FIGURE 2:**
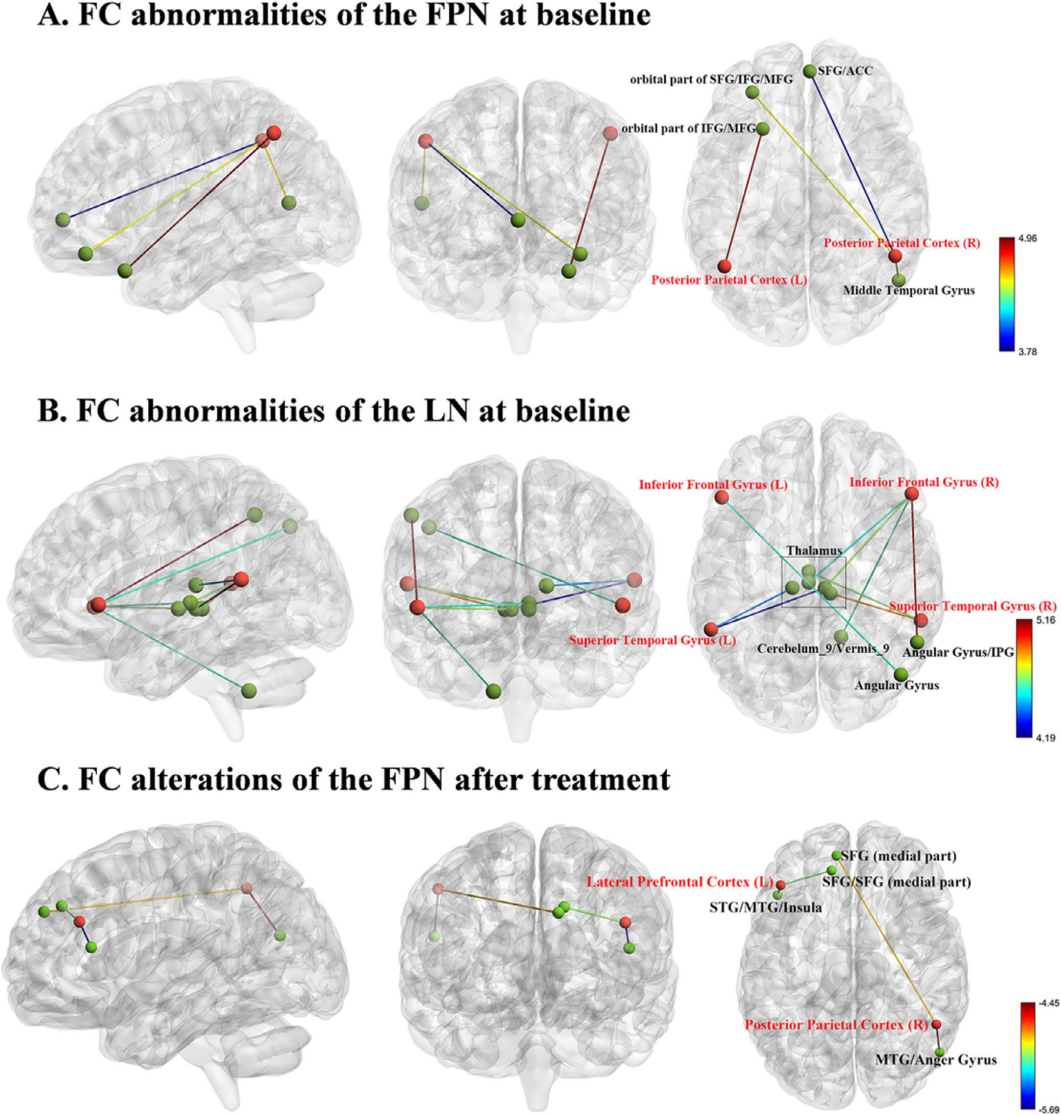
(A) FC abnormalities of the FPN at baseline in BD patients, with all observed FCs of FPN significantly increased compared to healthy controls, suggesting FPN hyperconnectivity. (B) FC abnormalities of the LN at baseline in BD patients, with all observed FCs of LN significantly increased compared to healthy controls, suggesting LN hyperconnectivity. (C) Posttreatment FC alterations in the FPN of BD patients, with all observed FCs significantly decreased, suggesting partial normalization after treatment. Red balls represent regions of interest, whereas green balls represent the brain regions connected to them. ACC, anterior cingulate cortex; FC, functional connectivity; FPN, fronto‐parietal network; IFG, inferior frontal gyrus; L, left; LN, language network; MFG, middle frontal gyrus; R, right; SFG, superior frontal gyrus.

Relative to HCs, BD patients showed significantly enhanced FC between different ROIs of the FPN and LN and the entire brain at baseline (Table 2). Regarding the FPN, the BD group exhibited higher FC between the left PPC (ROI3) and left IFG/MFG (both orbital part), as well as enhanced FC between the right PPC (ROI4) and right MTG, left SFG/IFG/MFG (all orbital part) and left SFG/ACC, in comparison to HCs. However, no significant difference in FC was detected in the bilateral LPFC (ROI1 and ROI2) between the two groups (Figure 2A). In the LN, patients with BD exhibited elevated FC between the left IFG (ROI5) and right angular gyrus (AG) and increased FC between the right IFG (ROI6) and right Cerebelum_9/Vermis_9, bilateral Thalamus, and right AG/inferior parietal gyrus (IPG). Increased FC between the bilateral STG (ROI7 and ROI8) and bilateral thalamus was also identified (Figure 2B). Compared to HCs, BD patients in the manic phase exhibited FC alterations consistent with those observed when analyzing the entire BD group (Table S7 and Figure S9).

**TABLE 2:** The changes in functional connectivity within the fronto‐parietal network and language network of BD patients at baseline compared to healthy controls.

ROIs	Brain regions	MNI (*x*, *y*, *z*)	*t*	Cluster size
*Fronto‐parietal network*				
Lateral prefrontal cortex (L)	/	/	/	/
Lateral prefrontal cortex (R)	/	/	/	/
Posterior parietal cortex (L)	L IFG (orbital part)/MFG (orbital part)	−24, 21, −24	4.96	85
Posterior parietal cortex (R)	R MTG	54, −66, 12	4.54	163
	L SFG (orbital part)/IFG (orbital part)/MFG (orbital part)	−30, 42, −15	4.51	159
	L SFG/ACC	3, 54, 3	3.78	58
*Language network*				
Inferior frontal gyrus (L)	R Angular Gyrus	48, −72, 42	4.59	55
Inferior frontal gyrus (R)	R Cerebellum_9/Vermis_9	15, −51, −42	4.62	130
	L Thalamus	−3, −21, 3	4.56	72
	R Thalamus	6, −24, 0	4.71	69
	R Angular Gyrus/IPG	57, −54, 48	5.16	63
Superior temporal gyrus (L)	L Thalamus	−12, −24, 12	4.44	100
	R Thalamus	6, −24, 0	4.19	64
Superior temporal gyrus (R)	L Thalamus	−3, −15, 0	4.68	73
	R Thalamus	9, −27, 0	4.91	71

Abbreviations: ACC, anterior cingulate cortex; IFG, inferior frontal gyrus; IPG, inferior parietal gyrus; L, left; MFG, middle frontal gyrus; MNI, Montreal Neurologic Institute; MTG, middle temporal gyrus; R, right; ROI, regions of interest; SFG, superior frontal gyrus.

### 3.4 FC Analyses Between BD Patients at Pretreatment and Posttreatment

After treatment, there were significantly reduced FCs between the FPN and other regions in individuals with BD, as depicted in Table 3 and Figure 2C. Specifically, patients with BD showed decreased FC between ROI1 and the left STG/MTG/insula/SFG/SFG (medial part), and lower FC between ROI2 and the left SFG (medial part), and right MTG/anger gyrus. Notably, some of the abnormal FCs at baseline were normalized after treatment.

**TABLE 3:** The alterations in functional connectivity of the fronto‐parietal network of BD patients after treatment.

ROIs	Brain regions	MNI (*x*, *y*, *z*)	*t*	Cluster size
*Fronto‐parietal network*				
Lateral prefrontal cortex (L)	L STG/MTG/insula	−45, 27, 15	−5.69	368
	L SFG/SFG (medial part)	−12, 42, 36	−5.07	157
Posterior parietal cortex (R)	L SFG (medial part)	−9, 51, 33	−4.81	56
	R MTG/angular gyrus	54, −69, 21	−4.45	55

Abbreviations: L, left; MNI, Montreal Neurologic Institute; MTG, middle temporal gyrus; R, right; ROI, regions of interest; SFG, superior frontal gyrus; STG, superior temporal gyrus.

### 3.5 Correlations Between FC and Clinical Features

At baseline, patients with BD showed FC between the ROI5 and the left IFG (triangular part, voxel size = 13 voxels, FWE‐corrected *p*‐value of cluster‐level = 0.001, and FWE‐corrected *p*‐value of peak level = 0.006), which was positively associated with N100, a type of ERP (*r* = 0.4531, *p* < 0.0001, Figure S10 and Table S8).

### 3.6 Neuroimaging–Transcription Association Analysis

By connecting gene expression profiles and case–control FC differences, genes related to FC alterations between different ROIs and the entire brain were identified, except for ROI7 (Table S9). Notably, genes related to FC changes were shared by several ROIs; these ROIs were all located in the FPN (Table 4). For instance, *MICAL1* was positively correlated with FC alterations based on ROI1–ROI3, whereas *ZNF768* showed positive correlations with FC alterations based on ROI2–ROI4. *EPCAM* was negatively correlated with FC changes among ROI2–ROI4. Due to the limited number of genes related to FCs of LN, only genes that positively or adversely correlated with FCs of FPN underwent separate GO and KEGG enrichment analyses, as presented in Figures S11–S14.

**TABLE 4:** The common seed‐to‐whole‐brain FC alteration‐related genes among ROIs.

ROIs	The names of common genes that showed positive correlations with FC alterations	The name of common genes that showed negative correlations with FC alterations	The proportion of common genes in FC alteration‐related genes
*Fronto‐parietal network*			
ROI 1 & 2	MICAL1		1/120 in ROI 1; 1/75 in ROI 2
		/	/
ROI 1 & 3	AARS2; ABCC5; ADAM11; ASGR1; CBY1; CEACAM19; CITED2; CLN3; COL19A1; CPNE9; DDX23; DHX32; DLST; DPH7; DUSP5; ELP1; FBXL20; FMR1; GEMIN7; GFPT2; GMPS; GSE1; JRK; KDM2B; KIAA2013; KLHL3; KMT2B; LOC100133331; MBTPS2; MED13L; MELTF‐AS1; MEX3C; MFHAS1; MICA; **MICAL1**; MLLT10; MRPL39; MXD1; NCLN; NOL10; NTM; NUP155; NXPH3; ORC2; PER1; PLEKHM1; POLN; PRKAB1; REPS1; RRS1; SELENOO; SMYD4; SUPV3L1; TARBP2; TTC17; ULK3; USP4; VPS13C; WDR81; YPEL4; ZNF133; ZNF148; ZNF563; ZNF605; ZNF776; ZSCAN31		66/120 in ROI 1; 66/219 in ROI 3
		BORCS5; C10orf82; CD84; CDC42; COMP; CPEB2; DRD2; GABRE; IL13RA1; KCTD10; LAMTOR1; LHX8; MAN1B1‐AS1; NDUFA2; PIGP; RAPSN; RESP18; SMIM14; TAGLN2	19/54 in ROI 1; 19/89 in ROI 3
ROI 1 & 4	/		/
		/	/
ROI 2 & 3	**MICAL1**; **ZNF768**		2/75 in ROI 2; 2/219 in ROI 3
		**EPCAM**; ITGA5; NDUFV3	3/132 in ROI 2; 3/89 in ROI 3
ROI 2 & 4	ANAPC4; CREBBP; ERH; N4BP2L1; RNF19A; SNAPC4; ZNF395; **ZNF768**		8/75 in ROI 2; 8/64 in ROI 4
		AADACL3; C11orf72; CALCOCO2; CCDC40; CD3D; CYP2B6; ENDOD1; **EPCAM**; IL9R; KDR; KRTAP4‐12; LOC541472; LRRC63; LTA; LTBR; PRM2; REXO2; SEPT10; SHOX; TMA16	20/132 in ROI 2; 20/131 in ROI 4
ROI 3 & 4	AP5Z1; **ZNF768**		
		**EPCAM**	1/89 in ROI 3; 1/131 in ROI 4

*Note:* For example, MICAL1 expression is positively correlated with FC values between ROI1 and the whole brain, and FC values between ROI2 and the whole brain. Therefore, MICAL1 is a common FC‐related gene for both ROI1 and ROI2. The bold text indicates the common genes shared across different ROIs.

Abbreviations: FC, functional connectivity; L, left; R, right; ROI, region of interest; ROI1, lateral prefrontal cortex (L); ROI2, lateral prefrontal cortex (R); ROI3, posterior parietal cortex (L); ROI4, posterior parietal cortex (R); ROI5, inferior frontal gyrus (L).

## 4 Discussion

Our study explored the resting‐state FC patterns of the FPN and LN and their evolution after treatment in BD patients. First, at baseline, BD patients present increased FPN–LN and FPN–PFG FCs, and elevated FC between the LN and bilateral thalamus, right AG, and right cerebellum. Second, after 3 months of treatment intervention, the increased FPN–LN and FPN–PFG FCs were partially normalized in follow‐up BD patients, and some of their clinical assessments significantly improved. Finally, FC alterations‐related genes and candidate pathophysiological processes for BD were identified.

### 4.1 At Baseline

At baseline, BD patients exhibited increased FC between the FPN and the LN (e.g., MTG and IFG), SFG/MFG/IFG (orbital part), and the ACC. FPN, as a cognitive hub, interacts with the rest of the brain and affects individual cognitive functioning [41]. For instance, in the resting state, greater DMN‐FPN networks and intra‐FPN connectivity were correlated with higher intelligence scores [42, 43]. These studies indicated a positive relationship between neural activity in FPN and cognitive functioning. BD patients often present neurocognitive impairments [44], and the observed hyperconnectivities of FPN at baseline could reflect reduced neural efficiency, which requires more neural resources to maintain its normal functions. Thus, the observed increased FCs in FPN might represent a compensatory mechanism for cognitive impairments in BD. The right FPN–left IFG hyperconnectivity in our study was consistent with the notion that the LN is left lateralized. Of note, hyperconnectivity between the FPN and the right MTG was found in our study. A possible interpretation is that the right MTG is involved in processing emotional and social information, which might explain the emotional and social dysfunction in BD. Increased activation of the right MTG during executive function tasks in BD patients was also observed by Tian et al. [45], indicating potential MTG involvement in the neural mechanisms of BD. On the other hand, we speculated that this hyperconnectivity could result from decreased activation in the left MTG, compensating for the loss of function in the left brain region, as observed in previous studies [17]. The orbital part of the frontal gyrus is a component of the PFC, which is involved in high‐level cognitive activities similar to the FPN. These activities include cognitive control, decision‐making processes, the regulation of social behaviors, and language function [46, 47]. Fleck et al. found aberrant prefrontal activity in bipolar mania, which might accompany attentional impairment [48]. Based on the similar function of FPN and the orbital part of the frontal gyrus in cognition, the enhanced FC between them might represent compensatory changes in response to cognitive impairments in BD.

Elevated FC between LN (IFG and STG) and bilateral thalamus, right AG/IPG, and right cerebellum was observed in our study. Investigations have demonstrated impairments in verbal associations, semantic contents, and discrepant prosody or speed of verbal production in BD patients [49, 50, 51]. The IPG plays a special role in proficient text reading and language learning [52, 53], and is deemed to be a part of LN. Several fMRI studies have shown abnormalities within LN related to various impaired language functions. The work of Hampson illustrated stronger FC between the left AG (part of IPG), left MTG, and left STG in skilled readers relative to poor readers [54]. Wang and his colleagues found that Chinese reading efficiency was positively correlated with the FC between the left inferior temporal gyrus (ITG) and left IPG [55]. Language abnormalities (e.g., switching and clustering abnormalities, and semantic overactivation) have been reported in BD patients across different mood states [56]. Therefore, we speculated that the increased IFG‐AG/IPG connections could explain dysfunction in language processing. However, these disturbances in LN might be influenced by different mood states of BD. In bipolar depressive patients, reduced FC within the left IFG, left MTG, and left AG was observed [57]. This hypoconnectivity within language regions might be attributed to thought retardation in depressive episodes, which was reversed after treatment [58]. Notably, in our study, the majority of the BD patients recruited were in manic episodes, and this might account for the hyperconnectivity within LN, which was opposite to that of depressed bipolar patients. Moreover, FC between the left IFG and the triangular part of IFG was positively correlated with N100. The left IFG is crucial to various language functions and is involved in prefrontal cognitive control [18]. The triangular part of the IFG, also part of Broca's area, is crucial for simultaneous language translation and may be involved in unconscious information processing. ERP provides temporal insights into stimulus/information processing, and N100 reflects early sensory processing [59]. The positive correlation between the FC and N100 might suggest that more cognitive resources are needed to process early perceptual input in BD patients. The thalamus is considered a relay station that connects and coordinates information pathways across the cortex. The thalamus may play a moderating role in language function by connecting areas that are crucial, similar to its role in sensory processing [60]. In research based on ERPs, the authors proposed that thalamic structures might be engaged in the analysis of syntactic and semantic parameters of presented sentences [61]. Because the communications between LN and other regions might be impaired in BD, the increased LN–thalamus FC observed in our study may represent a compensatory response to improve linguistic function. In addition to being essential for motor control, the cerebellum may also be involved in language, attention, and emotional regulation [62]. The work of Chen reported decreased spontaneous functional activity in the left MTG, extending to left STG and left cerebellum. Besides, the reduced FC between language regions and the cerebellum might be related to poor language skills in autism [63]. These results pointed to a potential role for the cerebellum in language functioning; hence, aberrant connections between the cerebellum and LN might help to explain the pathophysiology of language impairments in BD.

Compared to HCs, BD patients in the manic phase exhibited FC alterations consistent with those observed when analyzing the entire BD group. These results further confirmed that the increased FPN and LN FCs in BD patients might serve as a functional basis in the pathophysiology of BD. The observed increased FCs might represent a compensatory response to cognitive impairments in BD patients.

### 4.2 After Treatment

After 3 months of treatment, some of the baseline hyperconnectivity between FPN and LN, and with the frontal gyrus, was reduced. Moreover, clinical assessments of BD patients have changed significantly. Similarly, Spielberg et al. found hyperconnectivity in the network centered around the right amygdala in manic BD. These aberrant connections significantly reduced after 8 weeks of lithium treatment, which were proportional to symptom change [6]. Another systematic review reported baseline DMN and FPN hyperactivity in BD and MDD patients, which could be attenuated by cognitive treatments [64]. Based on previous results and our findings, the therapeutic intervention in BD patients appeared to have a normalizing effect, reducing susceptibility to disruption. These decreased FCs after treatment supported our proposals above that the increased FCs of FPN observed in BD patients might represent a compensatory response to cognitive impairments. After treatment, abnormal FCs between the left LPFC and the left STG/MTG/insula and SFG, despite no significant changes at baseline, were also observed, manifesting as hypoconnectivity between the FPN and LN and within the FPN. The pharmacotherapy of BD might help restore functional differentiation and improve neural efficiency. After pharmacotherapy, the brain gradually returned to an equilibrium state, thus reducing reliance on the overengaged FPN. Our findings underscored the crucial involvement of the FPN in the pharmacotherapy of BD patients. However, some abnormal FCs at baseline (e.g., abnormal FCs of the LN) did not recover, which could be attributed to the relatively small sample size and short intervention duration.

### 4.3 Potential Genetic Mechanisms in FC Changes

Using neuroimaging–transcription association analysis, several FC‐related genes shared by multiple ROIs in the FPN were identified. These common genes among ROIs might provide clues to underlying genetic mechanisms in BD. *MICAL1* encodes proteins involved in signal transduction, cell migration, and cytoskeletal remodeling. While most studies suggested its relationship with the advancement of cancer [65], a few also indicated that *MICAL1* may play a role in neural development following spinal cord injury [66], and the occurrence of lateral temporal epilepsy [67]. According to these investigations, the regulation of the oxidoreductase encoded by *MICAL1* plays a crucial part in these neural processes. *ZNF768*, which was positively correlated with FC changes, might take part in the regulation of gene expression; however, its precise roles and associated diseases have not been thoroughly studied [68]. *ZNF768* related to the FC of shared ROIs in the FPN raised the possibility of common transcriptional patterns in aberrant alterations in brain function. Apart from these, enrichment analyses revealed that the gene profiles were associated with pathways such as “immune receptor activity” and “oxidoreductase activity,” indicating potential candidate pathophysiological processes in BD etiology. Notably, our results pinpointed that transcriptional changes could account for most of the relationships with abnormal FCs of FPN in BD, providing a fresh viewpoint on the genetic mechanisms underlying BD.

### 4.4 Limitations

The current study has several shortcomings. First, due to the limited data of bipolar depressive patients, we only compared the baseline FCs between manic patients and HCs in the subsequent analysis. BD patients in different mood states might present distinct anomalous functional changes both at baseline and after treatment [8, 69]. However, the abnormal FCs observed in manic patients were consistent with the findings in the entire BD group, further validating our results. Second, the results might be influenced by a significant attrition rate due to the epidemic of COVID‐19 among BD patients who completed the follow‐up. However, no significant differences (see Figures S1–S8) were observed in the clinical and demographic data among all baseline patients (*n* = 77) and those patients who completed follow‐up (*n* = 38). This might reduce the confounding impact of the high attrition rate among follow‐up BD patients on our results. Finally, in the neuroimaging–transcription association analysis, the gene expression profiles from the AHBA were not collected from the same subjects as the fMRI data. Therefore, we may identify only genes with highly conserved expression profiles, potentially overlooking genes with larger interindividual transcriptional variability. Further validation should be conducted by correlating fMRI data with gene expression data from the same population.

## 5 Conclusions

In conclusion, the present study identified increased FPN and LN FCs in BD patients, which might represent a compensatory response to cognitive impairments. Longitudinal observation identified decreased FCs of FPN, indicating potential therapeutic mechanisms. Finally, FC alterations‐related genes and candidate pathophysiological processes associated with BD were discovered. From functional and genetic standpoints, these findings provided a better understanding of the pathophysiology underlying BD.

## Conflicts of Interest

The authors declare no conflicts of interest.

## Supporting information


Appendix S1

Table S9

## Data Availability

The data that support the findings of this study are available from the corresponding author upon reasonable request.
