# Supplementary material for: Fronto‐Parietal and Language Network Connectivity and Its Association With Gene Expression Profiles in Bipolar Disorder Before and After Treatment
Source: CNS Neurosci Ther. 2025 Feb 15;31(2):e70236. doi: 10.1111/cns.70236 (PMC11829113; doi:10.1111/cns.70236)
Supplement: Supplementary file 1 — Appendix S1 [file CNS-31-e70236-s001.docx]

**Supplementary materials**

1. **Method**

**2.3 Assessments**

Event-Related Potential data were obtained employing a myoelectric evoked potentiometer (Japanese Kohden MEB-9402C). Participants were directed to sit comfortably and exert focus. Electrode positioning adhered to the International Electroencephalogram Association's 10/20 standard. The ground was placed at the FPz hand center. The recording used the right ear M2 point, with Cz serving as the reference electrode. Electrode impedance was set at < 5 KΩ and the filter spanned 0.2–20 Hz. The analysis spanned 1,000 ms. Stimulation followed the conventional "Oddball" auditory mode, performing at a 1 time/s frequency, lasting 10 ms, and with a 5 μV sensitivity. Detection involved triggering and activating two systems, filtered 200 times via low-frequency and high-frequency filters. Non-target stimulus settings were 80% probability, 70 dB intensity, and 1000 Hz frequency, while target stimulus parameters were 20% probability, 90 dB intensity, and 2000 Hz frequency. Random interspersing of the two frequencies occurred, with each case repeating twice before averaging. Subjects received the target stimulus, and the non-target stimulus was excluded from the response. If a subject had fewer than 80% hits, the test was invalidated. N100, P200, N200, and P300 wave latencies were individually recorded.

**2.4 Imaging data acquisition and preprocessing**

The resting-state fMRI data were preprocessed in MATLABR2018b (http: //www. mathworks.com) with the SPM12 and RESTplus software ^1^. The first 10 images were excluded. Subsequent preprocessing steps included slice-timing correction, head motion correction, registration between T1-weighted and echo-planar imaging template (EPI) images, and normalization to the standardized Montreal Neurological Institute (MNI) EPI template with the voxel size of 3 mm × 3 mm × 3 mm. After that, a 4-mm fullwidth at half-maximum Gaussian kernel was applied for image smoothing. The images were then linearly detrended to mitigate the physiological noise. Some unwanted signals were regressed out, including signals from cerebrospinal fluid, white matter centered region, and Friston-24 head motion parameters obtained through rigid body correction. The global signal was not removed. Subsequently, band-pass filtering (0.01–0.08 Hz) was conducted. Subjects who showed more than 2 mm of displacement and more than 2° of angular motion in the x, y, or z axes were excluded. For longitudinal imaging preprocessing, we visually inspected the brain images of each patient at both time points to exclude any images with significant morphological differences. The preprocessing steps were applied consistently to both time points.

**2.7 Neuroimaging–transcription association analysis**

Abagen toolbox (version 0.1.1; <https://github.com/rmarkello/abagen>) was used to complete the first 6 processing steps to link gene expression sourced from AHBA and neuroimaging ^2^. First, we re-annotated the probes to acquire new physiological results, as the AHBA annotation tables may become outdated. To avoid the expression signal from the noise, probes showing poor correlation with RNA-seq measurements were eliminated. Subsequent processing steps included probe selection, remapping the sample to ALL-116 atlas, removing the inter-individual differences between the 6 donors, and gene filtering. Ultimately, a 15,633 ×116 (genes × regions) gene expression matrix was obtained.


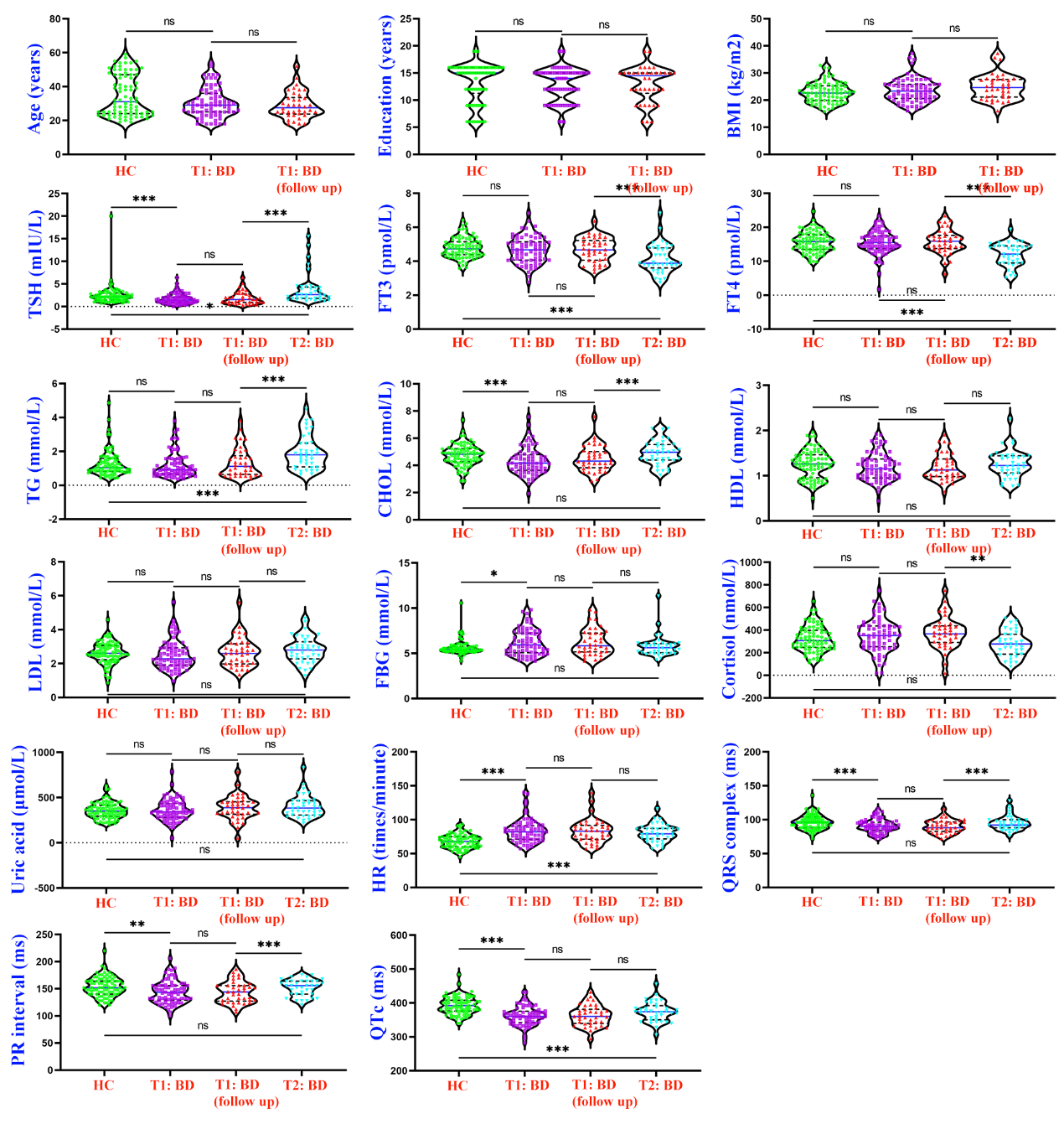


Figure S1: Between group differences in demography information and clinical variables. ns represents no difference; * represents p <0.05; ** represents p <0.01; *** represents p <0.001; **** represents p <0.0001. BMI= Body Mass Index; TSH=Thyroid Stimulating Hormone; FT3=Free Triiodothyronine; FT4=Free Thyroxine; TG=triglyceride; CHOL=Cholesterol; HDL=High Density Lipoprotein; LDL=Low Density Lipoprotein; FBG=Fasting Blood Glucose; HR=Heart Rate.


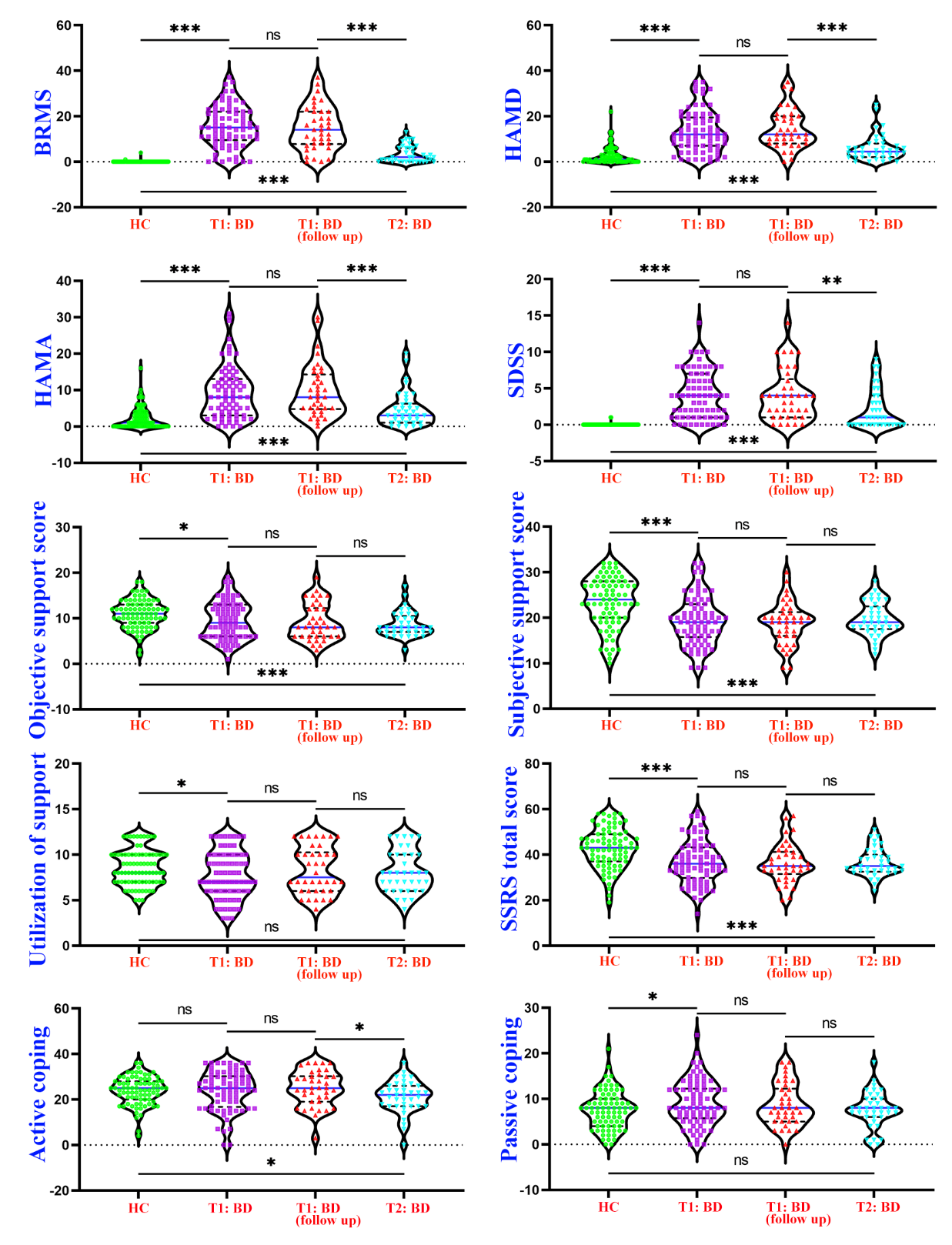


Figure S2: Between group differences in BRMS, HAMD, HAMA, SDSS, SSRS, and SCSQ. ns represents no difference; * represents p <0.05; ** represents p <0.01; *** represents p <0.001; **** represents p <0.0001. BRMS = Bech-Rafaelsen Mania Rating Scale; HAMD = Hamilton Depression Rating Scale; HAMA =Hamilton Anxiety Rating Scale; SDSS = Social Disability Screening Schedule; SSRS = Social Support Rating Scale; SCSQ = Simplified Coping Style Questionnaire.


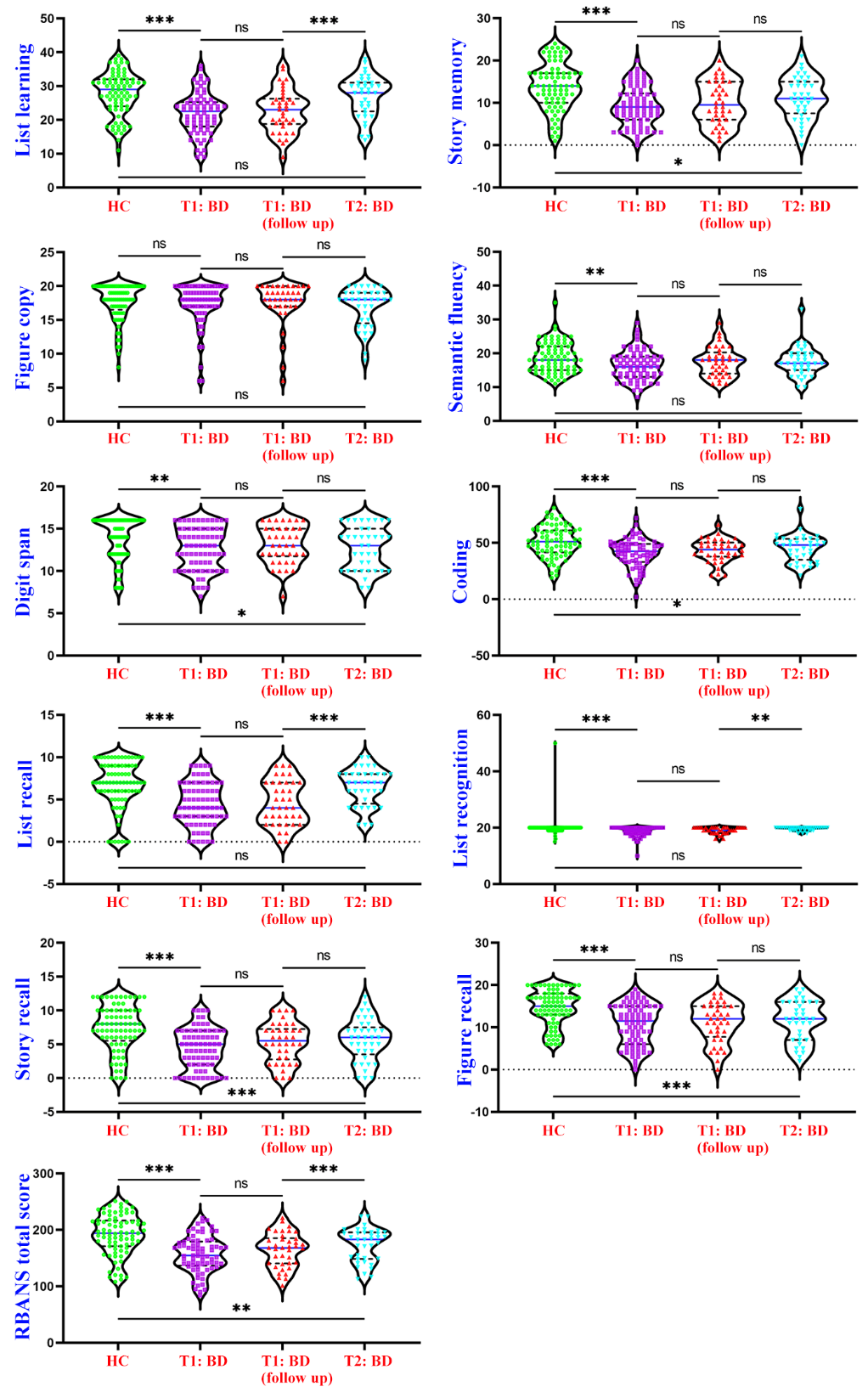


Figure S3: Between group differences in Repeatable Battery for the Assessment of Neuropsychological Status. ns represents no difference; * represents p <0.05; ** represents p <0.01; *** represents p <0.001; **** represents p <0.0001.


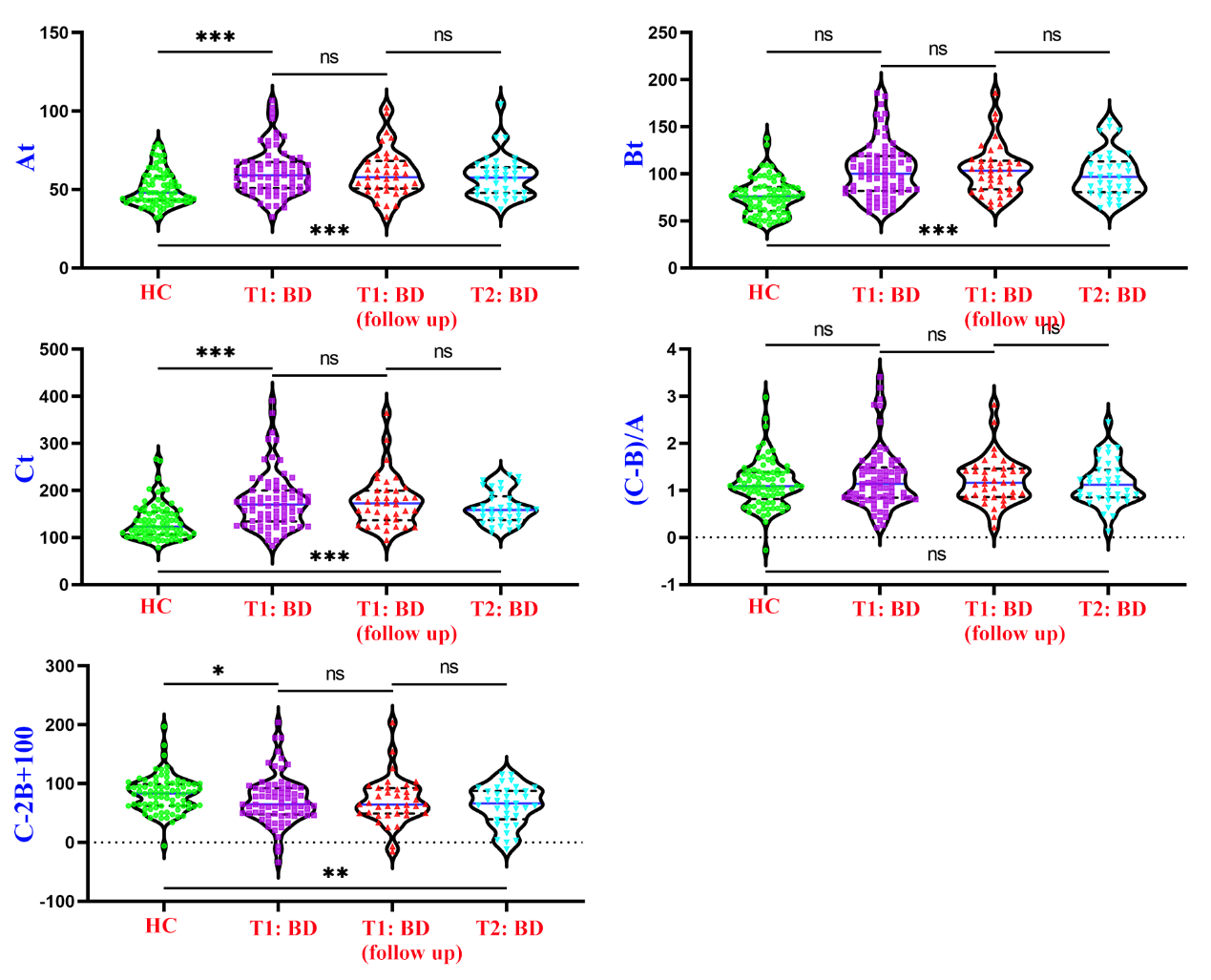


Figure S4: Between group differences in Stroop Color Word Test. ns represents no difference; * represents p <0.05; ** represents p <0.01; *** represents p <0.001; **** represents p <0.0001.


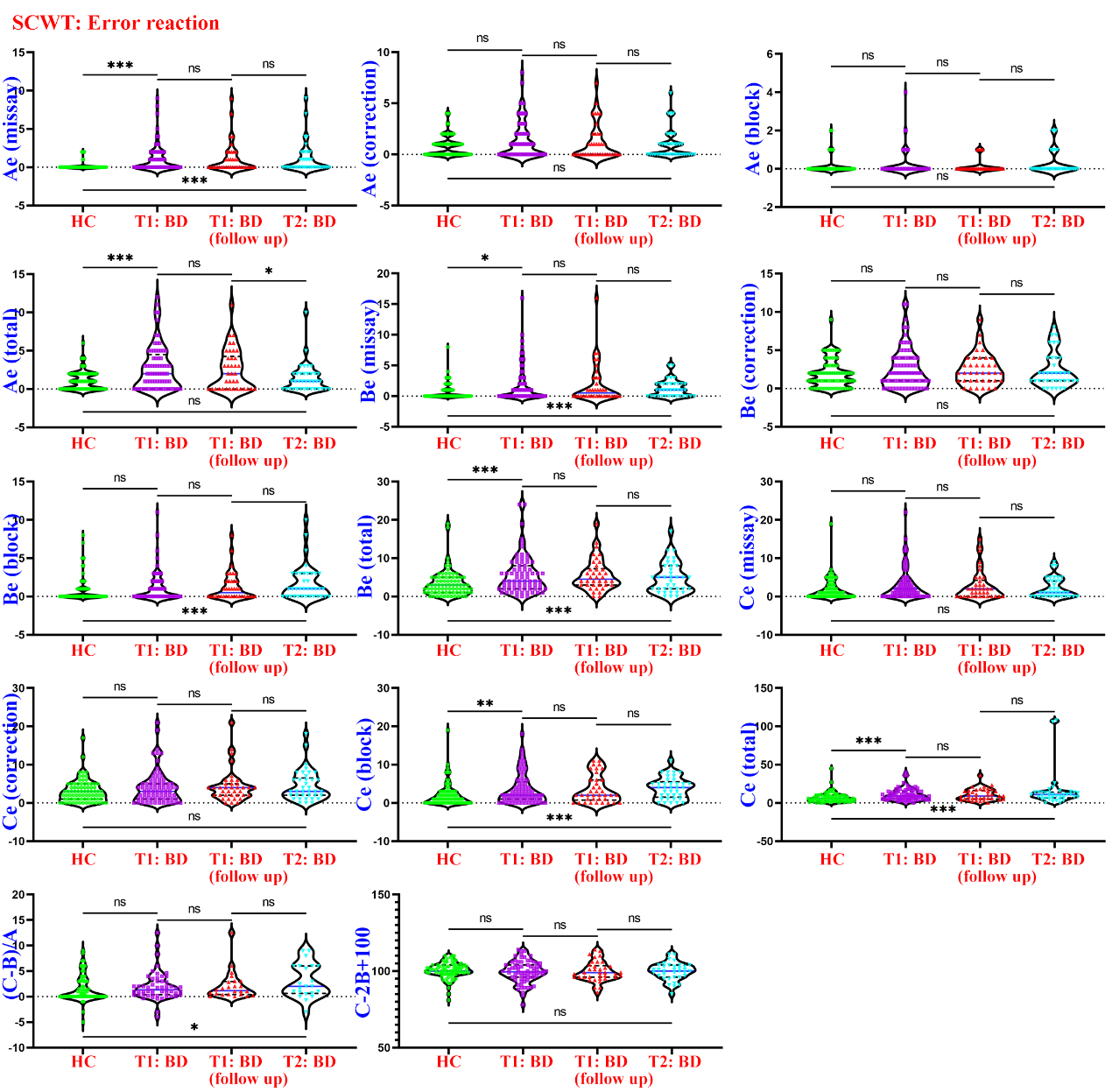


Figure S5: Between group differences in Error Reaction of Stroop Color Word Test. ns represents no difference; * represents p <0.05; ** represents p <0.01; *** represents p <0.001; **** represents p <0.0001.


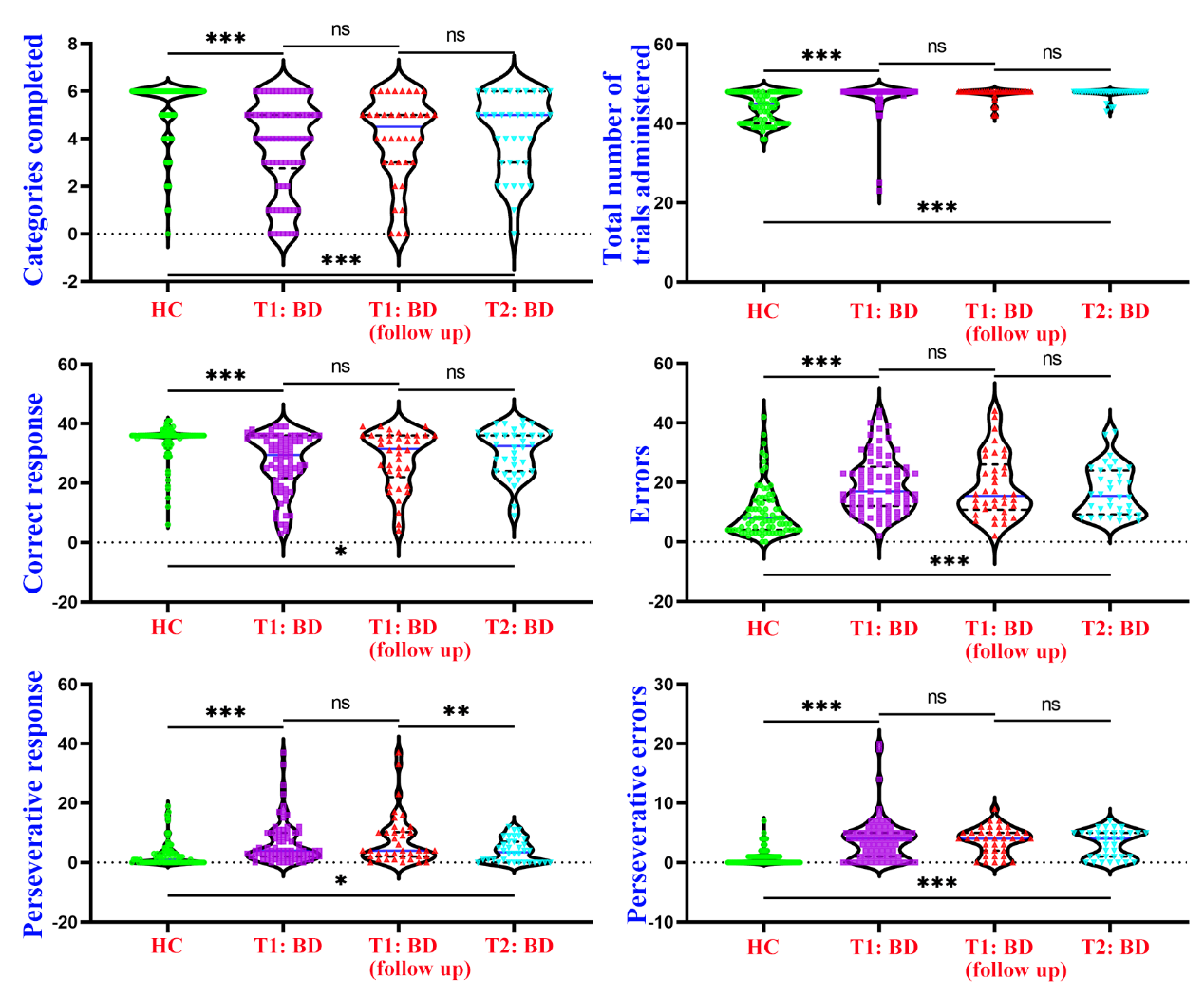


Figure S6: Between group differences in Wisconsin Card Sorting Test. ns represents no difference; * represents p <0.05; ** represents p <0.01; *** represents p <0.001; **** represents p <0.0001.


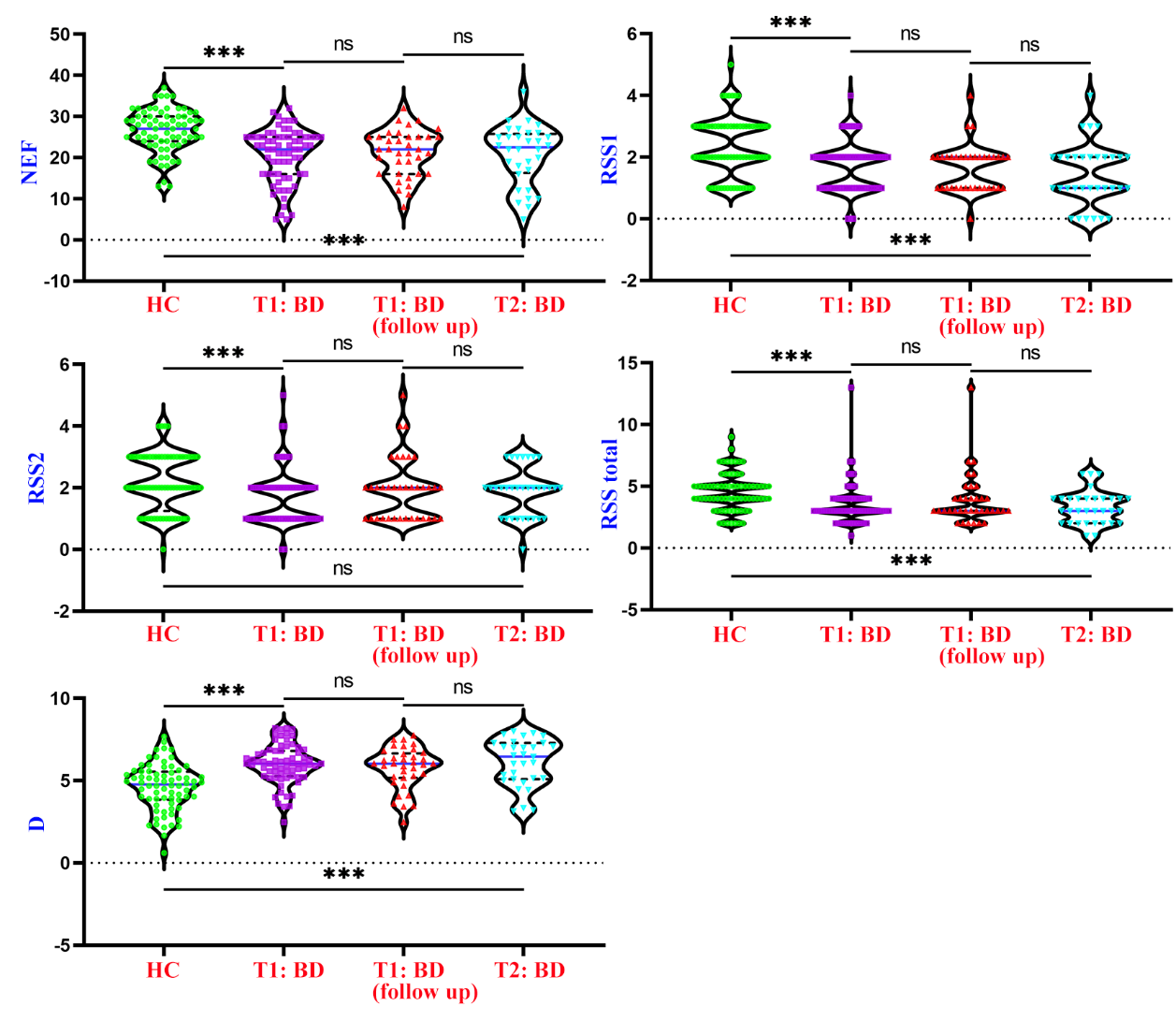


Figure S7: Between group differences in Exploratory Eye Movement. ns represents no difference; * represents p <0.05; ** represents p <0.01; *** represents p <0.001; **** represents p <0.0001. NEF = number of eye fixation; RSS = responsive search score; D=Discriminant analysis.


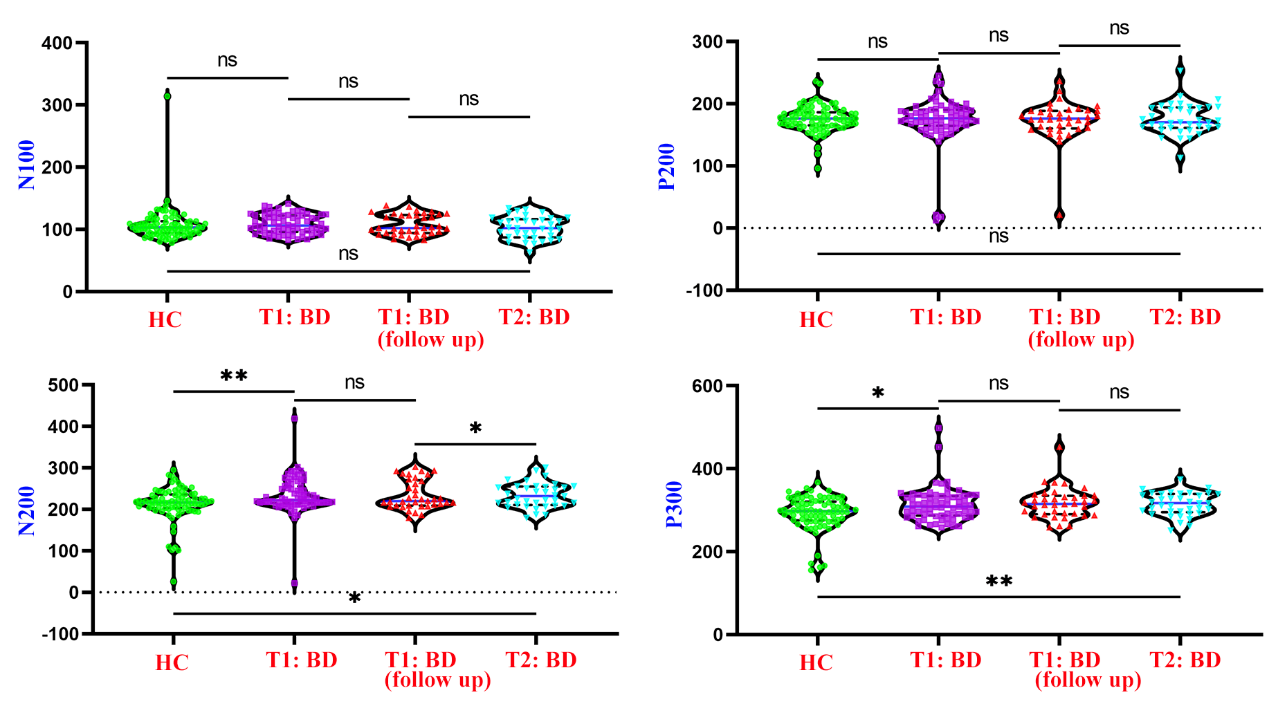


Figure S8: Between group differences in Event Related Potential. ns represents no difference; * represents p <0.05; ** represents p <0.01; *** represents p <0.001; **** represents p <0.0001.


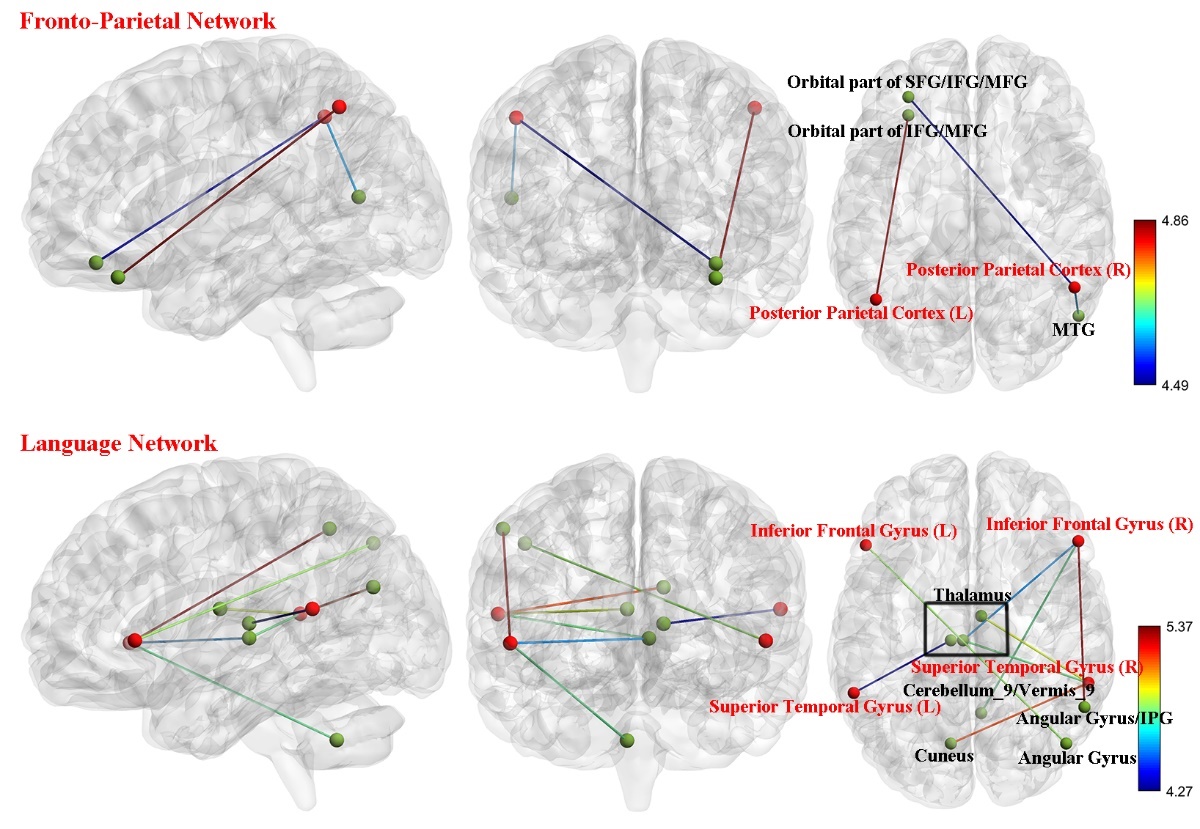


Figure S9: Functional connectivity abnormalities in the Fronto-Parietal Network and Language Network were observed in BD patients during a manic episode at baseline compared to healthy controls. The functional connectivity observed in manic BD patients was significantly increased compared to healthy controls. Red spheres indicate regions of interest (ROIs), while green spheres represent brain regions connected to these ROIs. BD = bipolar disorder; R = right; L = left; IFG = inferior frontal gyrus; MFG = middle frontal gyrus; MTG = middle temporal gyrus; SFG = superior frontal gyrus; IPG = inferior parietal gyrus.


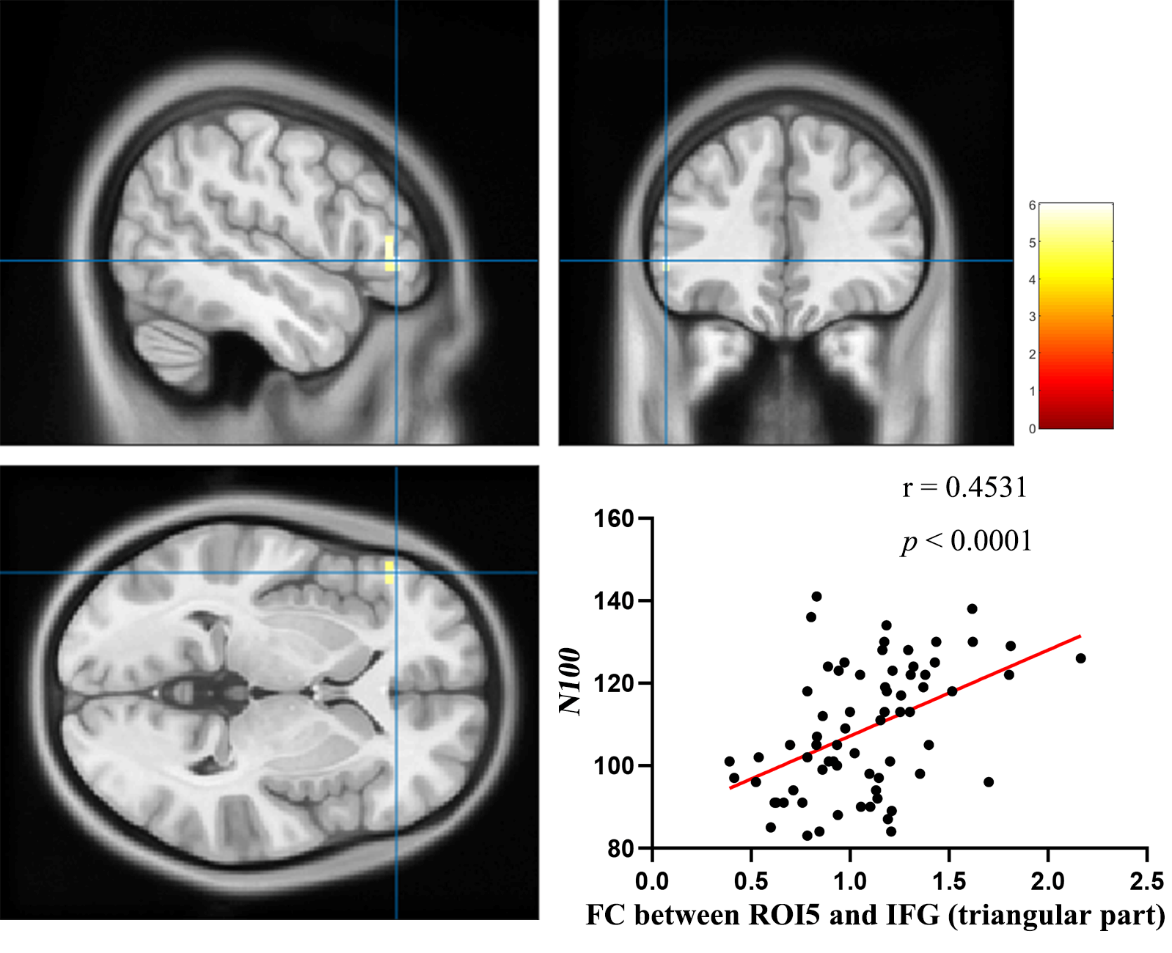


Figure S10. Correlation between FC of Language Network and clinical assessments in BD patients at baseline. FC = functional connectivity; ROI5 = left inferior frontal gyrus; IFG = inferior frontal gyrus; N100 is a type of event-related potential.


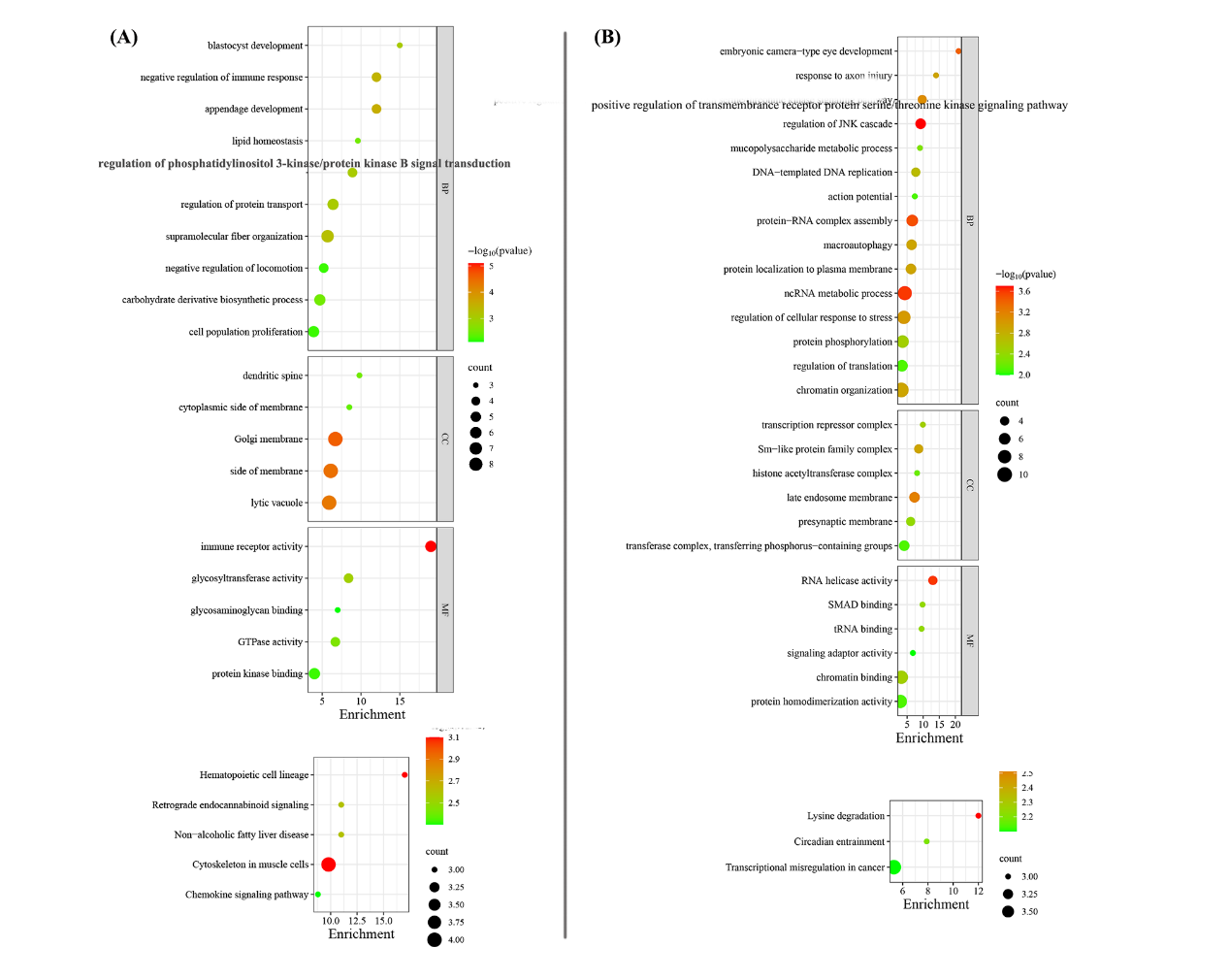


Figure S11. The two bubble diagrams depict the enrichment results of genes whose expression levels were negatively correlated with functional connectivity alterations (Part A) or positively correlated with functional connectivity alterations (Part B) from the left lateral prefrontal cortex to the rest of the brain regions.


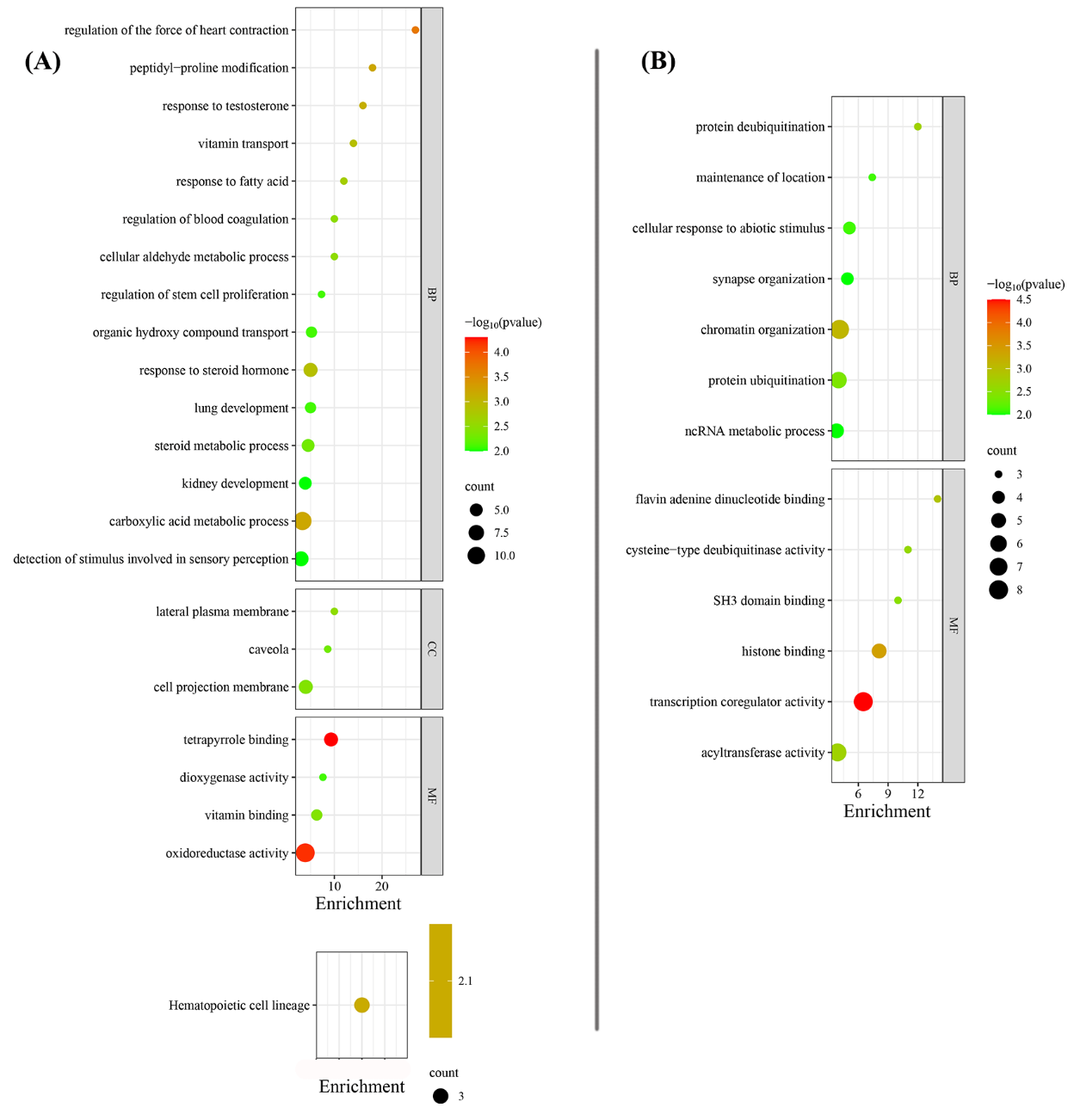


Figure S12. The two bubble diagrams depict the enrichment results of genes whose expression levels were negatively correlated with functional connectivity alterations (Part A) or positively correlated with functional connectivity alterations (Part B) from the right lateral prefrontal cortex to the rest of the brain regions.


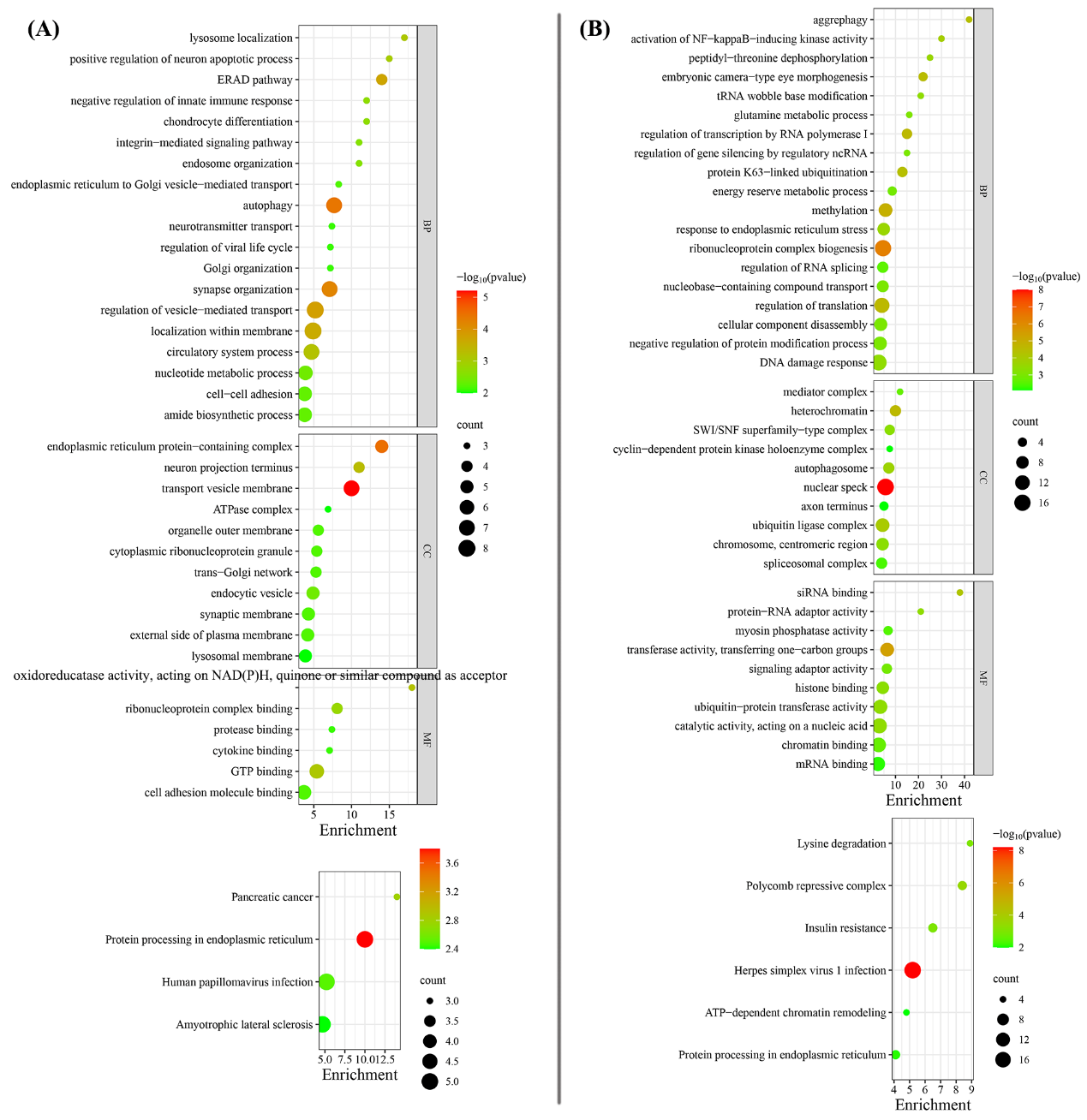


Figure S13. The two bubble diagrams depict the enrichment results of genes whose expression levels were negatively correlated with functional connectivity alterations (Part A) or positively correlated with functional connectivity alterations (Part B) from the left posterior parietal cortex to the rest of the brain regions.


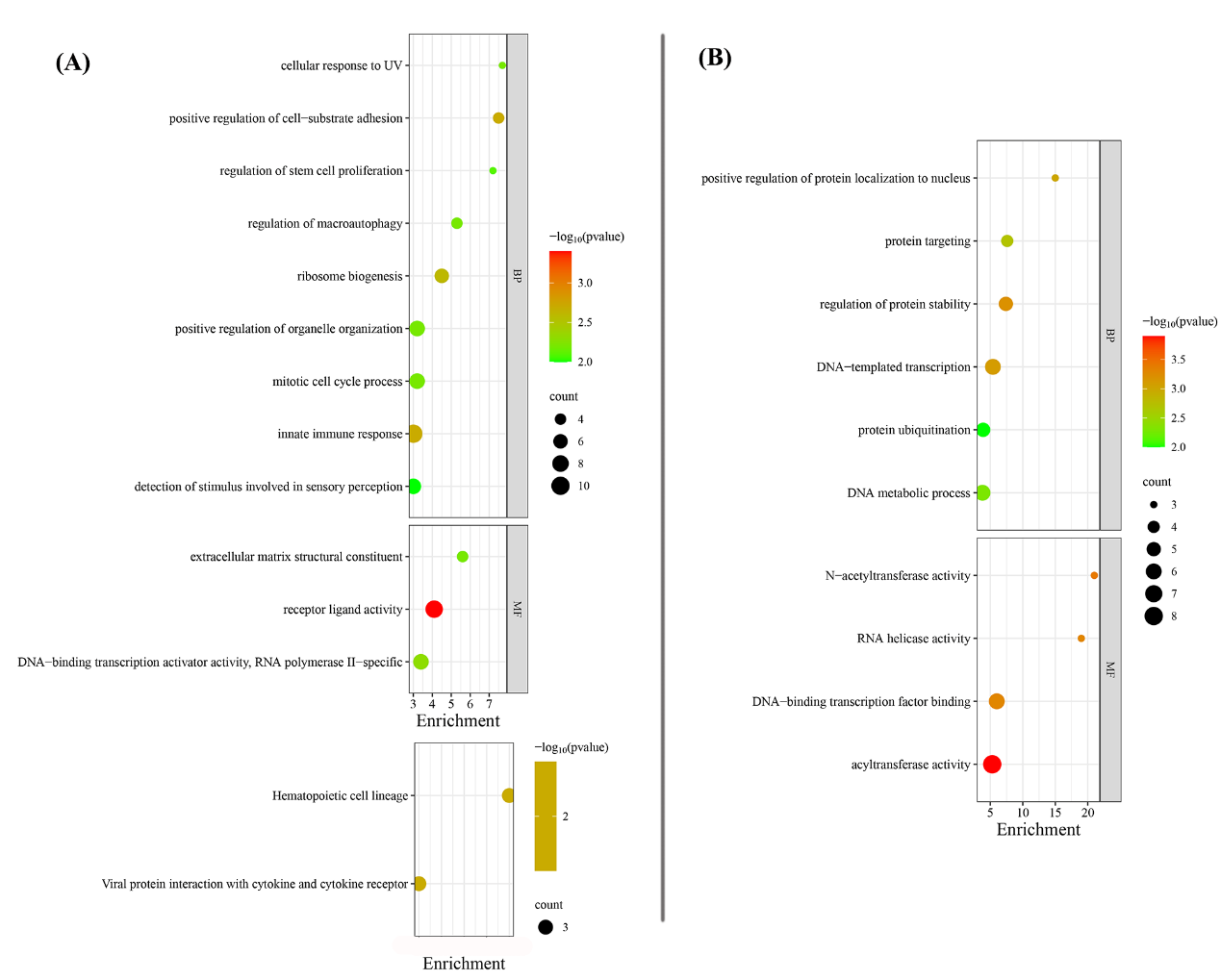


Figure S14. The two bubble diagrams depict the enrichment results of genes whose expression levels were negatively correlated with functional connectivity alterations (Part A) or positively correlated with functional connectivity alterations (Part B) from the right posterior parietal cortex to the rest of the brain regions.

Table S1. The coordinates of region of interests.

| Region of Interests (ROIs) | | MNI (x, y, z) |
| --- | --- | --- |
| Fronto-Parietal  Network | ROI1: Lateral Prefrontal Cortex (L) | -43 33 28 |
|  | ROI2: Lateral Prefrontal Cortex (R) | 41 38 30 |
|  | ROI3: Posterior Parietal Cortex (L) | -46 -58 49 |
|  | ROI4: Posterior Parietal Cortex (R) | 52 -52 45 |
| Language  Network | ROI5: Inferior Frontal Gyrus (L) | -51 26 2 |
|  | ROI6: Inferior Frontal Gyrus (R) | 54 28 1 |
|  | ROI7: (posterior) Superior Temporal Gyrus (L) | -57 -47 15 |
|  | ROI8: (posterior) Superior Temporal Gyrus (R) | 59 -42 13 |

MNI = Montreal Neurologic Institute; R = right; L = left.

Table S2. Comparison of patients and healthy controls in psychological status

| Variables | Patients (Mean ± SD, n=77) | Controls (Mean ± SD, n=83) | *p*-value |
| --- | --- | --- | --- |
| **BRMS** | 15.45±9.19 | 0.06±0.45 | <0.001^a^ |
| **HAMD** | 13.42±9.20 | 2.80±3.88 | <0.001^a^ |
| **HAMA** | 9.36±7.31 | 2.27±2.92 | <0.001^a^ |
| **SDSS** | 4.08±3.26 | 0.02±0.16 | <0.001^a^ |
| **SSRS** |  |  |  |
| Objective support score | 9.49±4.14 | 10.94±3.15 | 0.016^b^ |
| Subjective support score | 19.45±5.46 | 23.32±5.54 | <0.001^a^ |
| Utilization of support | 7.74±2.57 | 8.59±1.95 | 0.031^a^ |
| total score | 36.76±9.66 | 42.86±8.77 | <0.001^b^ |
| **SCSQ** |  |  |  |
| Active coping | 23.95±8.77 | 23.89±6.20 | 0.622^a^ |
| Passive coping | 9.11±5.03 | 7.57±4.14 | 0.041^a^ |

SD = Standard Deviation; BRMS = Bech-Rafaelsen Mania Rating Scale; HAMD = Hamilton Depression Rating Scale; HAMA =Hamilton Anxiety Rating Scale; SDSS = Social Disability Screening Schedule; SSRS = Social Support Rating Scale; SCSQ = Simplified Coping Style Questionnaire.

^a^ The p-values were obtained by a Mann-Whitney U test.

^b^ The *p*-values were obtained by two sample *t*-tests.

Table S3. Comparison of patients and healthy controls in cognitive status

| Tests | Patients (Mean ± SD, n=77) | Controls (Mean ± SD, n=83) | *p*-value |
| --- | --- | --- | --- |
| **RBANS** |  |  |  |
| List learning | 21.95±6.14 | 27.70±6.42 | <0.001^a^ |
| Story memory | 9.19±4.65 | 13.88±5.68 | <0.001^b^ |
| Figure copy | 17.38±3.06 | 17.64±2.70 | 0.533^a^ |
| Semantic fluency | 16.47±4.44 | 18.75±4.53 | 0.003^a^ |
| Digit span | 12.66±2.50 | 13.79±2.51 | 0.002^a^ |
| Coding | 40.73±12.73 | 50.88±13.78 | <0.001^b^ |
| List recall | 4.45±2.55 | 6.72±2.85 | <0.001^a^ |
| List recognition | 18.70±1.67 | 19.99±3.48 | <0.001^a^ |
| Story recall | 4.42±2.95 | 7.33±3.41 | <0.001^a^ |
| Figure recall | 10.64±4.83 | 14.67±4.18 | <0.001^a^ |
| Total score | 156.28±32.23 | 191.35±35.98 | <0.001^a^ |
| **SCWT** |  |  |  |
| At | 61.11±14.96 | 50.83±11.13 | <0.001^a^ |
| Bt | 103.44±29.43 | 76.04±18.86 | <0.001^a^ |
| Ct | 178.03±60.68 | 133.95±37.29 | <0.001^a^ |
| (C-B)/A | 1.24±0.64 | 1.15±0.50 | 0.675^a^ |
| C-2B+100 | 72.10±41.78 | 82.26±30.09 | 0.016^a^ |
| **Error reaction** |  |  |  |
| Ae (missay) | 1.14±1.84 | 0.15±0.50 | <0.001^a^ |
| Ae (correction) | 1.41±1.81 | 0.80±1.02 | 0.100^a^ |
| Ae (block) | 0.22±0.63 | 0.14±0.41 | 0.455^a^ |
| Ae (total) | 2.77±2.89 | 1.09±1.22 | <0.001^a^ |
| Be (missay) | 1.78±2.95 | 0.67±1.27 | 0.011^a^ |
| Be (correction) | 2.68±2.62 | 1.88±1.76 | 0.105^a^ |
| Be (block) | 1.18±1.95 | 0.80±1.62 | 0.076^a^ |
| Be (total) | 5.68±4.98 | 3.34±3.34 | 0.001^a^ |
| Ce (missay) | 3.03±4.18 | 1.84±2.75 | 0.089^a^ |
| Ce (correction) | 4.15±4.31 | 3.06±2.90 | 0.200^a^ |
| Ce (block) | 3.66±3.99 | 2.09±2.99 | 0.004^a^ |
| Ce (total) | 10.85±8.09 | 6.99±6.58 | <0.001^a^ |
| (C-B)/A | 1.99±2.76 | 1.51±2.55 | 0.053^a^ |
| C-2B+100 | 99.50±7.14 | 100.44±5.16 | 0.337^a^ |
| **WCST** |  |  |  |
| Categories completed | 3.70±1.94 | 5.20±1.40 | <0.001^a^ |
| Total number of trials administered | 46.84±4.07 | 44.01±3.78 | <0.001^a^ |
| Correct response | 26.99±9.74 | 33.90±6.24 | <0.001^a^ |
| Errors | 19.55±9.68 | 10.11±8.53 | <0.001^a^ |
| Perseverative response | 6.61±7.36 | 2.68±4.23 | <0.001^a^ |
| Perseverative errors | 3.81±3.73 | 0.80±1.35 | <0.001^a^ |
| **EEM** |  |  |  |
| NEF | 20.51±6.59 | 26.53±5.21 | <0.001^a^ |
| RSS1 | 1.68±0.80 | 2.35±0.95 | <0.001^a^ |
| RSS2 | 1.68±0.90 | 2.18±0.91 | <0.001^a^ |
| RSS total | 3.51±1.67 | 4.53±1.53 | <0.001^a^ |
| D | 6.00±1.22 | 4.60±1.42 | <0.001^b^ |
| **ERP** |  |  |  |
| N100 | 108.85±15.52 | 106.94±27.09 | 0.137^a^ |
| P200 | 172.83±38.96 | 175.07±20.85 | 0.600^a^ |
| N200 | 233.30±44.45 | 213.27±41.17 | 0.005^a^ |
| P300 | 312.91±39.50 | 292.93±41.31 | 0.019^a^ |

SD = Standard Deviation; RBANS = Repeatable Battery for the Assessment of Neuropsychological Status; SCWT=Stroop color word test; WCST = Wisconsin card sorting test; EEM = Exploratory eye movement; NEF = number of eye fixation; RSS = responsive search score; D=Discriminant analysis; ERP=Event related potential.

^a^ The p-values were obtained by a Mann-Whitney U test.

^b^ The *p*-values were obtained by two sample *t*-tests.

Table S4. Characteristics of patients who finished the follow-up.

| Variables | Pre-treatment (Mean ± SD, n=38) | Post-treatment (Mean ± SD, n=38) | *p* |
| --- | --- | --- | --- |
| Age (years) | 28.82±8.12 |  |  |
| Gender (male/female) | 11/27 |  |  |
| Years of education (years) | 12.97±3.34 |  |  |
| BMI (kg/m^2^) | 24.86±4.73 |  |  |
| TSH (mIU/L) | 1.90±1.44 | 3.83±3.41 | <0.001^a^ |
| FT3 (pmol/L) | 4.70±0.71 | 4.14±0.86 | 0.001^a^ |
| FT4 (pmol/L) | 15.87±3.65 | 11.95±3.35 | <0.001^b^ |
| TG (mmol/L) | 1.34±0.86 | 1.94±1.00 | <0.001^a^ |
| CHOL (mmol/L) | 4.50±0.95 | 4.91±0.89 | 0.001^b^ |
| HDL (mmol/L) | 1.19±0.30 | 1.25±0.31 | 0.260^b^ |
| LDL (mmol/L) | 2.67±0.86 | 2.80±0.78 | 0.072^a^ |
| FBG (mmol/L) | 6.26±1.49 | 5.66±1.48 | 0.049^a^ |
| Cortisol (nmol/L) | 378.48±149.11 | 278.99±115.65 | 0.002^b^ |
| Uric acid (μmol/L) | 387.46±134.41 | 402.78±122.98 | 0.464^a^ |
| HR (times/minute) | 82.43±17.26 | 80.41±12.99 | 0.889^a^ |
| QRS complex (ms) | 89.95±9.83 | 95.32±11.02 | <0.001^b^ |
| PR interval (ms) | 143.27±19.86 | 152.08±15.15 | 0.001^b^ |
| QTc (ms) | 363.46±30.11 | 374.03±29.42 | 0.074^b^ |

SD = Standard Deviation; BMI= Body Mass Index; TSH=Thyroid Stimulating Hormone; FT3=Free Triiodothyronine; FT4=Free Thyroxine; TG=triglyceride; CHOL=Cholesterol; HDL=High Density Lipoprotein; LDL=Low Density Lipoprotein; FBG=Fasting Blood Glucose; HR=Heart Rate.

^a^ The *p*-values were obtained by Wilcoxon signed-rank tests

^b^ The *p*-values were obtained by paired *t*-tests.

Table S5. Characteristics of patients who finished the follow-up.

| Variables | Pre-treatment (Mean ± SD, n=38) | Post-treatment (Mean ± SD, n=38) | *p* |
| --- | --- | --- | --- |
| **BRMS** | 14.76±9.68 | 3.34±3.70 | <0.001^a^ |
| **HAMD** | 14.43±8.55 | 6.08±5.80 | <0.001^a^ |
| **HAMA** | 9.74±7.22 | 4.74±4.75 | <0.001^a^ |
| **SDSS** | 4.11±3.52 | 2.18±2.73 | 0.005^a^ |
| **SSRS** |  |  |  |
| Objective support score | 9.16±3.99 | 8.65±2.78 | 0.518^a^ |
| Subjective support score | 18.84±4.71 | 19.68±3.71 | 0.269^b^ |
| Utilization of support | 8.32±2.56 | 8.14±2.31 | 0.636^a^ |
| total score | 36.32±8.57 | 36.46±6.14 | 0.851^b^ |
| **SCSQ** |  | ± |  |
| Active coping | 24.82±7.75 | 21.21±7.14 | 0.015^b^ |
| Passive coping | 8.76±4.62 | 7.63±3.93 | 0.192^b^ |

SD = Standard Deviation; BRMS = Bech-Rafaelsen Mania Rating Scale; HAMD = Hamilton Depression Rating Scale; HAMA =Hamilton Anxiety Rating Scale; SDSS = Social Disability Screening Schedule; SSRS = Social Support Rating Scale; SCSQ = Simplified Coping Style Questionnaire.

^a^ The *p*-values were obtained by Wilcoxon signed-rank tests

^b^ The *p*-values were obtained by paired *t*-tests.

Table S6. Characteristics of patients who finished the follow-up.

| Variables | Pre-treatment (Mean ± SD, n=38) | Post-treatment (Mean ± SD, n=38) | *p* |
| --- | --- | --- | --- |
| **RBANS** |  |  |  |
| List learning | 22.74±6.32 | 27.03±6.10 | <0.001^a^ |
| Story memory | 9.95±5.08 | 11.05±5.01 | 0.097^a^ |
| Figure copy | 17.61±3.19 | 16.86±2.95 | 0.141^b^ |
| Semantic fluency | 17.58±4.37 | 17.46±4.25 | 0.850^b^ |
| Digit span | 13.13±2.24 | 12.73±2.56 | 0.180^b^ |
| Coding | 43.08±10.24 | 45.00±11.80 | 0.171^a^ |
| List recall | 4.58±2.65 | 6.51±2.19 | <0.001^b^ |
| List recognition | 19.03±1.17 | 19.59±0.64 | 0.004^b^ |
| Story recall | 5.32±2.95 | 5.59±3.12 | 0.402^a^ |
| Figure recall | 11.11±4.73 | 11.51±4.82 | 0.503^a^ |
| Total scores | 164.11±30.43 | 173.35±29.75 | 0.001^a^ |
| **SCWT** |  |  |  |
| At | 60.47±15.37 | 58.14±13.32 | 0.148^a^ |
| Bt | 104.18±26.55 | 99.12±24.16 | 0.125^a^ |
| Ct | 177.20±54.14 | 164.18±34.14 | 0.213^b^ |
| (C-B)/A | 1.21±0.50 | 1.15±0.47 | 0.738^a^ |
| C-2B+100 | 70.65±39.27 | 62.42±33.34 | 0.678^b^ |
| **Error reaction** |  |  |  |
| Ae(missay) | 1.13±2.00 | 0.41±0.87 | 0.075^b^ |
| Ae(correction) | 1.21±1.76 | 0.86±1.36 | 0.139^b^ |
| Ae(block) | 0.13±0.34 | 0.30±0.62 | 0.184^b^ |
| Ae(total) | 2.47±2.69 | 1.41±1.89 | 0.037^b^ |
| Be(missay) | 1.84±3.19 | 1.24±1.34 | 0.889^b^ |
| Be(correction) | 2.47±2.06 | 2.35±2.25 | 0.635^b^ |
| Be(block) | 1.24±1.81 | 1.78±2.29 | 0.365^b^ |
| Be(total) | 5.63±4.09 | 4.86±4.02 | 0.315^a^ |
| Ce(missay) | 2.92±3.89 | 2.24±2.59 | 0.693^b^ |
| Ce(correction) | 4.34±4.21 | 4.57±4.03 | 0.952^b^ |
| Ce(block) | 3.61±3.61 | 3.92±2.79 | 0.693^b^ |
| Ce(total) | 10.89±7.49 | 15.22±22.91 | 0.658^b^ |
| (C-B)/A | 2.10±2.72 | 3.05±3.35 | 0.816^b^ |
| C-2B+100 | 99.68±6.54 | 99.80±5.78 | 0.918^b^ |
| **WCST** |  |  |  |
| Categories completed | 4.00±1.82 | 4.25±1.66 | 0.487^b^ |
| Total number of trials administered | 47.39±1.60 | 47.56±1.30 | 0.553^b^ |
| Correct response | 28.26±9.60 | 30.39±8.03 | 0.168^b^ |
| Errors | 18.55±10.60 | 17.11±8.30 | 0.300^b^ |
| Perseverative response | 7.55±8.59 | 4.00±3.86 | 0.008^b^ |
| Perseverative errors | 3.71±2.24 | 3.14±2.30 | 0.203^b^ |
| **EEM** |  |  |  |
| NEF | 21.17±5.64 | 20.72±7.29 | 0.911^a^ |
| RSS1 | 1.64±0.76 | 1.44±1.01 | 0.313^b^ |
| RSS2 | 1.94±1.01 | 1.88±0.79 | 0.733^b^ |
| RSS total | 3.86±2.06 | 3.28±1.33 | 0.244^b^ |
| D | 5.77±1.27 | 6.09±1.43 | 0.405^b^ |
| **ERP** |  |  |  |
| N100 | 107.60±16.20 | 102.87±18.02 | 0.160^b^ |
| P200 | 173.08±32.56 | 175.54±24.77 | 0.385^b^ |
| N200 | 234.62±35.09 | 233.97±29.40 | 0.896^b^ |
| P300 | 316.78±36.55 | 314.22±27.71 | 0.906^b^ |

SD = Standard Deviation; RBANS = Repeatable Battery for the Assessment of Neuropsychological Status; SCWT=Stroop color word test; WCST = Wisconsin card sorting test; EEM = Exploratory eye movement; NEF = number of eye fixation; RSS = responsive search score; D=Discriminant analysis; ERP=Event related potential.

^a^ The *p*-values were obtained by paired *t*-tests.

^b^ The *p*-values were obtained by Wilcoxon signed-rank tests

Table S7. At baseline, the functional connectivity within the Fronto-Parietal Network and Language Network in BD patients experiencing a manic episode compared to healthy controls.

| ROIs | Brain Regions | MNI  (x, y, z) | T values | Cluster size |
| --- | --- | --- | --- | --- |
| Fronto-Parietal Network | | | | |
| Lateral Prefrontal Cortex (L) | / | / | / | / |
| Lateral Prefrontal Cortex (R) | / | / | / | / |
| Posterior Parietal Cortex (L) | L IFG (orbital part)/MFG (orbital part) | -30 33 -21 | 4.86 | 57 |
| Posterior Parietal Cortex (R) | R MTG | 54 -66 12 | 4.59 | 181 |
|  | L SFG (orbital part)/IFG (orbital part)/MFG (orbital part) | -30 42 -15 | 4.49 | 112 |
| Language Network | | | | |
| Inferior Frontal Gyrus (L) | R Angular Gyrus | 48 -72 42 | 4.85 | 91 |
| Inferior Frontal Gyrus (R) | R Cerebellum_9/Vermis_9 | 6 -57 -39 | 4.77 | 159 |
|  | R Angular Gyrus/IPG | 57 -54 48 | 5.37 | 85 |
|  | L Thalamus | -3 -21 3 | 4.55 | 65 |
| Superior Temporal Gyrus (L) | L Thalamus | -9 -21 9 | 4.27 | 86 |
| Superior Temporal Gyrus (R) | R/L Cuneus | -9 -72 24 | 5.16 | 116 |
|  | R Thalamus | 9 -27 0 | 4.91 | 71 |
|  | L Thalamus | -3 -15 0 | 4.68 | 73 |

ROI = regions of interest; MNI = Montreal Neurologic Institute; R = right; L = left; IFG = inferior frontal gyrus; MFG =middle frontal gyrus; MTG = Middle Temporal Gyrus; SFG = superior frontal gyrus; IPG = inferior parietal gyrus.

Table S8. The correlation between the functional connectivity of ROIs and N100.

| ROIs | Brain Regions | MNI  (x, y, z) | T values | Cluster size |
| --- | --- | --- | --- | --- |
| Language Network | | | | |
| Inferior Frontal Gyrus (L) | L IFG (triangular part) | -51 36 0 | 4.17 | 13 |

ROI = regions of interest; MNI = Montreal Neurologic Institute; L = left; IFG = inferior frontal gyrus.

**References**

1. Jia X-Z, Wang J, Sun H-Y, Zhang H, Liao W, Wang Z *et al.* RESTplus: an improved toolkit for resting-state functional magnetic resonance imaging data processing. *Science Bulletin* 2019; **64**(14)**:** 953-954.

2. Arnatkeviciute A, Fulcher BD, Fornito A. A practical guide to linking brain-wide gene expression and neuroimaging data. *Neuroimage* 2019; **189:** 353-367.
