# Supplementary material for: Fronto‐Parietal and Language Network Connectivity and Its Association With Gene Expression Profiles in Bipolar Disorder Before and After Treatment
Source: CNS Neurosci Ther. 2025 Feb 15;31(2):e70236. doi: 10.1111/cns.70236 (PMC11829113; doi:10.1111/cns.70236)
Supplement: Supplementary file 2 — Table S9 [file CNS-31-e70236-s002.pdf]

Table S9. Genes associated with functional connectivity alterations.

ROI1			ROI2		
Gene	rho	p	Gene	rho	p
AARS2	0.439411	8.03E-07	AADACL3	-0.45603	2.68E-07
ABCC5	0.441272	7.12E-07	ADM	-0.45761	2.41E-07
ACVR1B	0.459955	2.05E-07	AK2	-0.49144	2.12E-08
ADAM11	0.505406	7.19E-09	ALDH3B1	-0.44348	6.17E-07
AMER3	0.418002	3.03E-06	ANAPC4	0.455455	2.79E-07
ARHGDIB	-0.44602	5.23E-07	ANKRD13C	0.488462	2.66E-08
ASGR1	0.419148	2.83E-06	ANKRD18A	-0.43658	9.62E-07
B3GAT2	-0.4226	2.29E-06	AQP7P1	-0.42173	2.42E-06
BMPER	0.433952	1.14E-06	ASB16	-0.42292	2.25E-06
BORCS5	-0.46411	1.54E-07	ASPHD2	0.429598	1.49E-06
BRD9	0.439961	7.75E-07	ASXL1	0.44589	5.27E-07
C10orf82	-0.46939	1.07E-07	AXIN2	0.439343	8.06E-07
C1GALT1	-0.44657	5.04E-07	BBC3	0.446275	5.14E-07
CAMK2B	0.419167	2.82E-06	BCAS1	-0.46874	1.12E-07
CBY1	0.421104	2.51E-06	BCLAF1	0.432774	1.22E-06
CCL19	-0.41911	2.83E-06	BCO2	-0.45132	3.68E-07
CD84	-0.46103	1.91E-07	BCORP1	-0.46509	1.44E-07
CDC42	-0.41743	3.13E-06	BOP1	0.417396	3.14E-06
CDK9	0.431096	1.36E-06	BRCC3	0.429106	1.54E-06
CEACAM19	0.479088	5.32E-08	C11orf72	-0.41929	2.80E-06
CELSR3	0.446377	5.11E-07	C14orf132	0.417477	3.13E-06
CHST11	-0.42377	2.14E-06	C14orf177	-0.43631	9.79E-07
CITED2	0.459136	2.17E-07	C1orf35	0.446532	5.05E-07
CLCA4	-0.43064	1.40E-06	C5orf42	0.431989	1.29E-06
CLN3	0.438938	8.27E-07	C5orf58	-0.43897	8.25E-07
COL19A1	0.489491	2.46E-08	CALCOCO2	-0.42993	1.46E-06
COMP	-0.47005	1.02E-07	CBLL1	0.485052	3.43E-08
CPEB2	-0.42706	1.75E-06	CCDC160	-0.44499	5.59E-07
CPNE9	0.491194	2.16E-08	CCDC40	-0.45021	3.97E-07
CSF2RA	-0.44736	4.79E-07	CCNG2	0.472639	8.46E-08
CYC1	0.428985	1.55E-06	CD320	-0.41868	2.91E-06
DCN	-0.422	2.38E-06	CD3D	-0.4584	2.29E-07
DDX23	0.429627	1.49E-06	CDCA3	-0.48397	3.71E-08
DDX46	0.437051	9.33E-07	CES4A	0.431147	1.35E-06
DHX32	0.418148	3.00E-06	CHAF1B	0.44742	4.77E-07
DLST	0.421447	2.46E-06	CHRM5	-0.42844	1.60E-06
DNAJB12	0.42812	1.64E-06	CORO1C	-0.41757	3.11E-06
DNAJC3	-0.42032	2.64E-06	COX7A2L	-0.42154	2.45E-06
DPH7	0.465664	1.38E-07	CPT1A	-0.43305	1.20E-06
DRD2	-0.446	5.23E-07	CREBBP	0.417731	3.08E-06
DTX3	0.424091	2.10E-06	CRY2	0.478156	5.69E-08
DUSP5	0.454411	2.99E-07	CXCL6	-0.42859	1.59E-06
EIF2B2	0.423269	2.20E-06	CXXC1	0.419326	2.80E-06
ELF4	-0.42438	2.06E-06	CYP11B1	-0.44238	6.63E-07
ELP1	0.434402	1.10E-06	CYP2B6	-0.4345	1.10E-06
EPC2	0.457202	2.48E-07	CYS1	0.439428	8.02E-07
EVI2B	-0.41911	2.83E-06	DGCR5	0.423539	2.17E-06
FAM160B2	0.51993	2.21E-09	DHFR	-0.44865	4.40E-07
FBXL20	0.462065	1.78E-07	DLAT	-0.45287	3.32E-07
FBXO21	0.420093	2.67E-06	EIF2AK1	-0.43543	1.03E-06
FCHO2	0.458383	2.29E-07	EMC10	-0.49552	1.56E-08
FMR1	0.45425	3.03E-07	EMILIN2	-0.44451	5.77E-07
FRMD8	0.43609	9.92E-07	ENDOD1	-0.45342	3.20E-07
GABRE	-0.43745	9.10E-07	EPCAM	-0.47062	9.77E-08
GALC	-0.42088	2.55E-06	ERG	-0.43557	1.03E-06
GCNA	0.487895	2.77E-08	ERH	0.476253	6.53E-08

GEMIN7	0.48422	3.65E-08
GFPT2	0.468735	1.12E-07
GMPS	0.451232	3.70E-07
GNB3	0.421723	2.42E-06
GNG8	-0.44291	6.41E-07
GSE1	0.480007	4.97E-08
HERPUD1	0.419332	2.80E-06
HLA-DQB1	-0.48554	3.31E-08
HLA-DRB1	-0.46384	1.57E-07
IFFO2	0.431423	1.33E-06
IL13RA1	-0.45995	2.05E-07
ITIH4	0.433306	1.18E-06
JRK	0.4467	5.00E-07
KCNJ11	0.422992	2.24E-06
KCTD10	-0.43406	1.13E-06
KDM2B	0.433883	1.14E-06
KIAA2013	0.46313	1.65E-07
KLHL3	0.493988	1.75E-08
KMT2B	0.423123	2.22E-06
LAMTOR1	-0.42636	1.82E-06
LHX8	-0.43244	1.25E-06
LILRB4	-0.42855	1.59E-06
LINC00473	0.484981	3.45E-08
LINC01480	-0.43318	1.19E-06
LOC10013333	0.454484	2.98E-07
LRRC66	0.46433	1.52E-07
MAN1B1-AS1	-0.44173	6.91E-07
MAPK8IP3	0.479499	5.16E-08
MAPKBP1	0.42927	1.52E-06
MBTPS2	0.42481	2.00E-06
MED13L	0.449683	4.11E-07
MELTF-AS1	0.43321	1.19E-06
MEN1	0.440806	7.34E-07
MEX3C	0.428359	1.61E-06
MFHAS1	0.5177	2.66E-09
MICA	0.499205	1.17E-08
MICAL1	0.417768	3.07E-06
MLLT10	0.446754	4.98E-07
MREG	0.451186	3.72E-07
MRGBP	0.417679	3.09E-06
MRPL39	0.438834	8.33E-07
MXD1	0.467913	1.18E-07
MYBPC1	-0.4224	2.32E-06
NCLN	0.543653	2.86E-10
NDUFA2	-0.42542	1.93E-06
NOL10	0.48916	2.52E-08
NPC2	-0.42601	1.86E-06
NPPC	0.447584	4.72E-07
NREP	0.43587	1.01E-06
NRL	-0.42177	2.41E-06
NTM	0.504926	7.47E-09
NUP155	0.421031	2.52E-06
NXPH3	0.452262	3.46E-07
OARD1	-0.47024	1.00E-07
ORC2	0.429004	1.55E-06
ORC4	0.430119	1.44E-06
PALMD	-0.43406	1.13E-06
PARP14	-0.42789	1.66E-06

FAM136A	-0.44186	6.86E-07
FAM215A	-0.47178	9.00E-08
FAM218A	0.432351	1.26E-06
FAM27E2	0.427219	1.73E-06
FBXO38	0.428764	1.57E-06
FHIT	-0.42208	2.37E-06
FKBP4	-0.45516	2.85E-07
FUNDC1	-0.44848	4.45E-07
GET4	0.443096	6.33E-07
GGT3P	-0.41807	3.02E-06
GLRX3	-0.41943	2.78E-06
GYPC	-0.44388	6.01E-07
H1FNT	-0.44522	5.51E-07
HDGF	-0.44066	7.41E-07
HHIP-AS1	-0.42839	1.61E-06
HMGCS2	-0.48805	2.74E-08
HOXB13	-0.45194	3.53E-07
IL10RB	-0.47558	6.85E-08
IL9R	-0.46387	1.57E-07
ING4	0.439547	7.96E-07
ITGA5	-0.43241	1.25E-06
JADE3	-0.44464	5.72E-07
KANK4	-0.44951	4.15E-07
KCNK15	-0.48189	4.33E-08
KCNK5	-0.44245	6.60E-07
KDM5B	0.488501	2.65E-08
KDR	-0.41727	3.17E-06
KHDRBS1	0.421137	2.51E-06
KIF21A	-0.42254	2.30E-06
KLF14	-0.4216	2.44E-06
KRTAP10-10	-0.43744	9.10E-07
KRTAP4-12	-0.41931	2.80E-06
LEF1-AS1	-0.44694	4.92E-07
LEFTY1	0.422478	2.31E-06
LGALS3BP	-0.41801	3.03E-06
LHFPL5	-0.43335	1.18E-06
LILRA1	-0.42002	2.68E-06
LINC00626	-0.44405	5.94E-07
LLPH	-0.43534	1.04E-06
LOC100233156	-0.45368	3.14E-07
LOC101927596	-0.43639	9.73E-07
LOC105373876	-0.43856	8.48E-07
LOC541472	-0.43492	1.07E-06
LRFN3	0.46467	1.48E-07
LRFN4	0.447812	4.65E-07
LRRC63	-0.49387	1.76E-08
LTA	-0.42266	2.29E-06
LTBR	-0.44617	5.18E-07
MAFG-AS1	-0.42142	2.47E-06
MB21D2	0.418976	2.86E-06
MICAL1	0.42991	1.46E-06
MPIG6B	-0.42341	2.18E-06
MTTP	-0.44805	4.57E-07
N4BP2L1	0.460937	1.92E-07
NAALADL2	-0.42251	2.31E-06
NCOA6	0.445725	5.33E-07
NDUFV3	-0.4182	2.99E-06
NEURL3	0.509279	5.28E-09

PCDHA11	0.419617	2.75E-06
PER1	0.421623	2.43E-06
PGP	0.420739	2.57E-06
PIGP	-0.49262	1.94E-08
PLEKHM1	0.490083	2.35E-08
PNPLA4	-0.45195	3.53E-07
POLN	0.438615	8.45E-07
POLR3A	0.41987	2.71E-06
PRAG1	0.473014	8.24E-08
PRKAB1	0.442921	6.40E-07
QKI	-0.44128	7.12E-07
RAI2	0.418871	2.87E-06
RAPSN	-0.42842	1.60E-06
RARA	0.436309	9.78E-07
REPS1	0.427005	1.75E-06
RESP18	-0.49779	1.31E-08
RHOC	-0.43023	1.43E-06
RRS1	0.41844	2.95E-06
SBDS	-0.44465	5.72E-07
SDR16C5	0.419117	2.83E-06
SELENOO	0.418713	2.90E-06
SEMA3C	-0.4202	2.65E-06
SHISA8	0.417718	3.08E-06
SLC43A1	0.443313	6.24E-07
SMIM14	-0.47813	5.70E-08
SMOC1	-0.43906	8.21E-07
SMYD4	0.417168	3.18E-06
SREBF1	-0.43326	1.19E-06
ST3GAL2	0.424591	2.03E-06
SUPV3L1	0.435959	1.00E-06
SYNDIG1L	-0.47439	7.47E-08
SYNE1	0.426041	1.86E-06
TAF15	0.425072	1.97E-06
TAF5	0.417268	3.16E-06
TAGLN2	-0.4384	8.56E-07
TARBP2	0.419824	2.71E-06
TEAD3	-0.42662	1.79E-06
TMLHE	0.436193	9.86E-07
TRAM1L1	-0.43825	8.65E-07
TRMT9B	0.450859	3.80E-07
TSPAN5	0.462215	1.76E-07
TTC17	0.446065	5.21E-07
TTN	-0.46022	2.02E-07
ULK3	0.459544	2.11E-07
URGCP	0.438085	8.74E-07
USP4	0.437135	9.28E-07
VPS13C	0.417379	3.14E-06
WDR81	0.510742	4.70E-09
YPEL4	0.463042	1.66E-07
ZDHHC3	0.430242	1.43E-06
ZFHX3	-0.4311	1.36E-06
ZFPM2	0.431376	1.34E-06
ZNF133	0.431857	1.30E-06
ZNF148	0.446857	4.95E-07
ZNF324	0.423034	2.23E-06
ZNF563	0.42353	2.17E-06
ZNF605	0.447788	4.65E-07
ZNF655	0.442779	6.46E-07

NFKBIL1	0.455874	2.71E-07
NKX3-1	-0.43559	1.02E-06
NOS3	-0.43797	8.80E-07
NOXA1	0.446044	5.22E-07
NPHS2	-0.4322	1.27E-06
NR2C2AP	0.510655	4.73E-09
NSD3	0.425785	1.89E-06
NUP93	-0.41815	3.00E-06
OGFOD1	-0.43112	1.36E-06
OLMALINC	-0.41855	2.93E-06
OR10A2	-0.42385	2.13E-06
OR2H1	-0.42036	2.63E-06
OR2T6	-0.45988	2.07E-07
OR2W5	-0.4417	6.92E-07
OR4D6	-0.46466	1.48E-07
OR5L2	-0.4468	4.97E-07
OTUD1	0.450338	3.93E-07
OTUD6A	-0.45178	3.57E-07
PA2G4	0.44877	4.36E-07
PCLO	0.428383	1.61E-06
PER2	0.43252	1.24E-06
PHF12	-0.44341	6.20E-07
PHKB	-0.42271	2.28E-06
POLR2A	-0.45095	3.78E-07
PPIC	-0.43741	9.12E-07
PPOX	0.418384	2.96E-06
PRM2	-0.4376	9.01E-07
PRR13	-0.45191	3.54E-07
PTOV1	0.456412	2.62E-07
PTPDC1	-0.43105	1.36E-06
RBM5	0.438121	8.72E-07
REXO2	-0.41888	2.87E-06
RFX3	0.472232	8.71E-08
RHPN1	-0.41877	2.89E-06
RNF126	-0.42713	1.74E-06
RNF19A	0.454013	3.07E-07
RTN4R	0.42006	2.68E-06
SCAP	0.487843	2.78E-08
SCGB1A1	-0.44847	4.45E-07
SCN2A	0.457339	2.46E-07
SEPT10	-0.45444	2.99E-07
SERPINA6	-0.42513	1.97E-06
SETMAR	-0.52553	1.39E-09
SFN	-0.427	1.75E-06
SHOX	-0.47511	7.09E-08
SLC16A1	-0.42726	1.72E-06
SLC19A3	-0.44293	6.40E-07
SLC22A18AS	-0.41888	2.87E-06
SLC46A1	-0.46404	1.55E-07
SLC8A2	0.45138	3.67E-07
SLFN5	-0.44923	4.23E-07
SMAD7	-0.42621	1.84E-06
SNAPC4	0.465942	1.36E-07
SPEG	0.42161	2.44E-06
SSB	-0.42861	1.59E-06
SSTR3	-0.4278	1.67E-06
SUGT1P3	0.421291	2.48E-06
SYDE1	-0.44661	5.03E-07

ZNF776	0.440603	7.43E-07
ZSCAN31	0.461008	1.91E-07

TAL1	-0.46626	1.33E-07
TARBP1	0.419188	2.82E-06
TBXAS1	-0.42853	1.59E-06
TCTN3	-0.43734	9.16E-07
TMA16	-0.42528	1.95E-06
TMEM119	-0.42502	1.98E-06
TMEM189	-0.4426	6.54E-07
TMEM240	0.442503	6.57E-07
TMOD1	0.418953	2.86E-06
TOR4A	-0.43972	7.87E-07
TSPAN8	-0.47232	8.65E-08
TTC39A	-0.44094	7.27E-07
TUBGCP6	0.421598	2.44E-06
UHRF2	0.459342	2.14E-07
USF3	0.459495	2.12E-07
USP30	0.451242	3.70E-07
USP32P1	0.432854	1.22E-06
UTP6	0.461933	1.79E-07
VASH2	-0.44931	4.21E-07
VPREB3	-0.46992	1.03E-07
VPS13B	0.503782	8.18E-09
WDR59	0.439393	8.03E-07
WFDC10B	-0.4197	2.73E-06
ZBTB24	0.464935	1.46E-07
ZBTB26	-0.44351	6.16E-07
ZC3HAV1L	-0.42099	2.53E-06
ZNF208	-0.46852	1.13E-07
ZNF395	0.424789	2.01E-06
ZNF485	-0.45531	2.82E-07
ZNF623	0.424593	2.03E-06
ZNF682	0.426961	1.76E-06
ZNF707	-0.4212	2.50E-06
ZNF768	0.43157	1.32E-06
ZNF829	0.445218	5.51E-07
ZNF830	0.441408	7.06E-07

ROI3			ROI4		
Gene	rho	p	Gene	rho	p
AARS2	0.43055	1.41E-06	AADACL3	-0.44614	5.19E-07
ABCC5	0.443187	6.29E-07	ALPP	-0.45453	2.97E-07
ACSL4	-0.46722	1.24E-07	ANAPC4	0.476386	6.47E-08
ADAM11	0.444432	5.80E-07	AP4S1	0.422328	2.33E-06
ADAMTS7	-0.42668	1.79E-06	AP5Z1	0.457561	2.42E-07
ADGRG6	-0.42915	1.53E-06	ARL9	0.434222	1.12E-06
AGAP5	0.455673	2.75E-07	ART5	-0.42493	1.99E-06
AGO2	0.424861	2.00E-06	ATAD3C	-0.41812	3.01E-06
AIDA	0.494611	1.67E-08	ATL3	-0.42463	2.03E-06
AKAP17A	0.480157	4.92E-08	ATP10B	-0.47734	6.04E-08
AKT2	0.441218	7.14E-07	B3GNT7	-0.42703	1.75E-06
ALKBH1	0.440507	7.48E-07	BAG6	0.421455	2.46E-06
AMMECR1L	0.444974	5.60E-07	BANF1	-0.44995	4.03E-07
ANKHD1	0.439642	7.91E-07	BCL2L13	-0.46505	1.44E-07
ANKZF1	0.435955	1.00E-06	BGN	-0.46855	1.13E-07
AP5Z1	0.421697	2.42E-06	BMF	-0.46699	1.26E-07
ARIH2	0.496184	1.48E-08	BMP10	-0.47016	1.01E-07
ASGR1	0.475978	6.66E-08	BRD8	0.460252	2.01E-07
ASIC2	-0.44085	7.32E-07	BRPF3	0.434687	1.08E-06
ATAD3B	0.429755	1.48E-06	C11orf72	-0.46197	1.79E-07
ATF6	-0.42347	2.18E-06	C11orf95	0.424008	2.11E-06
ATG4B	0.418291	2.98E-06	C1QBP	-0.42415	2.09E-06
ATL2	0.432707	1.23E-06	CALCA	-0.41875	2.90E-06
ATP2C1	-0.46225	1.75E-07	CALCOCO2	-0.42447	2.05E-06
ATP6V1D	-0.51102	4.59E-09	CASP8	-0.42292	2.25E-06
BAZ1B	0.455915	2.70E-07	CASZ1	-0.43565	1.02E-06
BAZ2A	0.478096	5.71E-08	CCDC40	-0.4357	1.02E-06
BBS10	0.426987	1.75E-06	CCL25	-0.46486	1.46E-07
BORCS5	-0.44	7.73E-07	CCNB3	-0.48926	2.50E-08
BRICD5	0.458437	2.28E-07	CD3D	-0.41976	2.73E-06
BRWD3	0.440757	7.36E-07	CD47	0.422731	2.28E-06
C10orf82	-0.46831	1.15E-07	CENPK	-0.44877	4.36E-07
C4orf48	-0.45378	3.12E-07	CLUAP1	0.447681	4.69E-07
CAD	0.425514	1.92E-06	COL1A2	-0.4663	1.32E-07
CALCOCO1	0.457118	2.49E-07	COQ7	0.423454	2.18E-06
CALML4	0.421716	2.42E-06	COX4I2	-0.42102	2.52E-06
CBY1	0.455127	2.85E-07	CREBBP	0.417707	3.08E-06
CCDC86	-0.42055	2.60E-06	CSTF3	0.447031	4.89E-07
CCNL1	0.465775	1.37E-07	CTDNEP1	0.430259	1.43E-06
CD84	-0.44424	5.87E-07	CTNNA3	-0.46742	1.22E-07
CDC25B	0.458098	2.33E-07	CWC15	-0.45883	2.22E-07
CDC42	-0.51799	2.60E-09	CYP2B6	-0.48579	3.25E-08
CDHR3	0.421528	2.45E-06	DDX42	0.473073	8.20E-08
CDK11A	0.442314	6.66E-07	DDX51	0.426141	1.85E-06
CDK5RAP3	0.439961	7.75E-07	DEFB124	-0.41908	2.84E-06
CEACAM19	0.425791	1.89E-06	DEFB132	-0.44516	5.53E-07
CITED2	0.469416	1.06E-07	DEFB135	-0.44442	5.80E-07
CLK2	0.441545	7.00E-07	DHX33	0.426933	1.76E-06
CLN3	0.433395	1.18E-06	DKC1	-0.41751	3.12E-06
CMIP	-0.42212	2.36E-06	DMAP1	0.448231	4.52E-07
CNPPD1	0.476593	6.37E-08	DNM1P46	-0.4376	9.01E-07
CNPY4	0.439342	8.06E-07	DOCK9	-0.43383	1.14E-06
COL11A2	0.438077	8.74E-07	DPRX	-0.42977	1.48E-06
COL19A1	0.447388	4.78E-07	DPY19L1P1	0.440807	7.34E-07
COMP	-0.47094	9.55E-08	EIF5AL1	-0.44666	5.01E-07
COQ3	0.41816	3.00E-06	ENDOD1	-0.41778	3.07E-06

CPEB2	-0.44018	7.64E-07
CPNE9	0.478481	5.56E-08
CROCCP3	0.465975	1.35E-07
CRYGS	0.443175	6.29E-07
CYTL1	-0.43398	1.13E-06
DAB2IP	0.459694	2.09E-07
DAP3	-0.48537	3.35E-08
DDX23	0.515805	3.11E-09
DERL3	-0.46938	1.07E-07
DHX32	0.476463	6.43E-08
DIP2C	0.437662	8.98E-07
DLST	0.447096	4.87E-07
DMWD	-0.43029	1.43E-06
DMXL1	0.426014	1.86E-06
DPH7	0.506828	6.43E-09
DRD2	-0.50074	1.04E-08
DUSP5	0.434283	1.11E-06
E2F6	0.441533	7.00E-07
ECHDC1	-0.4236	2.16E-06
EDC3	0.429878	1.47E-06
EIF3M	-0.41834	2.97E-06
ELP1	0.453174	3.25E-07
ELP6	0.501627	9.69E-09
ENOX2	-0.44509	5.55E-07
EPCAM	-0.42121	2.50E-06
ESPL1	-0.43389	1.14E-06
EZH1	0.444686	5.70E-07
FAM126A	-0.42116	2.50E-06
FAM174B	-0.4697	1.04E-07
FAM193A	0.436501	9.67E-07
FAM76B	0.446504	5.06E-07
FBRS	0.427498	1.70E-06
FBXL20	0.438966	8.26E-07
FBXO15	-0.4302	1.44E-06
FEM1C	0.48971	2.42E-08
FMR1	0.449526	4.15E-07
FNBP4	0.47321	8.12E-08
GABPB1-IT1	0.444928	5.61E-07
GABRE	-0.43004	1.45E-06
GASAL1	-0.53496	6.16E-10
GEMIN7	0.46907	1.09E-07
GFPT2	0.447119	4.86E-07
GMPS	0.467478	1.22E-07
GRN	-0.43509	1.06E-06
GSE1	0.437701	8.95E-07
GTF3C2	0.491767	2.07E-08
GYS1	0.462343	1.74E-07
HDGFL1	-0.4541	3.06E-07
HENMT1	-0.46774	1.20E-07
HIPK1	0.489937	2.38E-08
IL13RA1	-0.43257	1.24E-06
IL2RB	-0.42329	2.20E-06
ILF3	0.433018	1.20E-06
ING3	0.42054	2.60E-06
INO80	0.427498	1.70E-06
INPP5E	0.427021	1.75E-06
INTS6-AS1	0.452067	3.50E-07
ISG15	-0.43957	7.95E-07
EPCAM	-0.48215	4.25E-08
EPHA3	0.437263	9.21E-07
ERH	0.425053	1.98E-06
ERP29	-0.43321	1.19E-06
EXOC1	-0.43311	1.20E-06
FAM114A2	0.430735	1.39E-06
FFAR1	-0.45005	4.01E-07
FLT3LG	-0.44764	4.70E-07
GATD1	0.421036	2.52E-06
GDF7	-0.43065	1.40E-06
GOLGA6L10	0.461263	1.88E-07
GPR25	-0.433	1.21E-06
HMGB2	0.436794	9.49E-07
HMHB1	-0.45622	2.65E-07
HNF1A-AS1	-0.42387	2.12E-06
HRH4	-0.43068	1.39E-06
HTATSF1	-0.42117	2.50E-06
HYDIN	0.442537	6.56E-07
IFNL2	-0.49939	1.15E-08
IFT88	-0.44208	6.76E-07
IL9R	-0.42072	2.57E-06
INTS13	-0.44192	6.83E-07
KBTBD4	0.420498	2.61E-06
KCNQ1DN	-0.42643	1.82E-06
KCTD7	-0.41775	3.08E-06
KDM2A	-0.43257	1.24E-06
KDR	-0.44429	5.85E-07
KLHL18	0.427202	1.73E-06
KRT15	-0.44958	4.14E-07
KRT78	-0.44179	6.89E-07
KRTAP23-1	-0.45745	2.44E-07
KRTAP4-12	-0.45477	2.92E-07
LAMB2P1	0.420913	2.54E-06
LARGE2	-0.43011	1.45E-06
LILRP2	-0.42704	1.75E-06
LINC01144	-0.47836	5.61E-08
LINC01351	0.444521	5.77E-07
LINC01993	-0.42024	2.65E-06
LOC541472	-0.4493	4.21E-07
LPIN3	-0.43601	9.97E-07
LRRC63	-0.42885	1.56E-06
LTA	-0.4577	2.40E-07
LTBR	-0.46908	1.09E-07
LYPD3	-0.41849	2.94E-06
LYPLA2	0.427352	1.71E-06
MAK16	-0.43797	8.80E-07
MARCH11	0.429328	1.52E-06
MFAP4	-0.41786	3.05E-06
MIOX	-0.43452	1.10E-06
MIR205HG	-0.41812	3.01E-06
MLLT6	0.441049	7.22E-07
MRFAP1L1	0.428394	1.61E-06
MRGPRX4	-0.42888	1.56E-06
MRPL13	-0.46665	1.29E-07
MUC1	-0.458	2.35E-07
MUC2	-0.44865	4.40E-07
MUC4	-0.42597	1.87E-06
N4BP2L1	0.451156	3.72E-07

ISY1	0.426233	1.84E-06	NAT14	0.495	1.62E-08
ITGA5	-0.42481	2.00E-06	NDUFA4L2	-0.42553	1.92E-06
JRK	0.468909	1.10E-07	NEDD4	-0.42256	2.30E-06
KCTD10	-0.56645	3.41E-11	NEURL4	0.45367	3.15E-07
KDM2B	0.436152	9.88E-07	NIFK	-0.42573	1.90E-06
KHDC4	0.465583	1.39E-07	NOC4L	-0.42157	2.44E-06
KIAA2013	0.433722	1.15E-06	NUDT19	-0.46437	1.52E-07
KLHL3	0.454719	2.93E-07	OR11A1	-0.41744	3.13E-06
KMT2B	0.458571	2.26E-07	OR1J4	-0.42332	2.20E-06
KMT5B	0.481422	4.48E-08	OR2B11	-0.43958	7.94E-07
KYAT3	-0.41818	3.00E-06	OR4D2	-0.42319	2.21E-06
LAMA5	0.439554	7.95E-07	OR52W1	-0.45156	3.63E-07
LAMTOR1	-0.42043	2.62E-06	PAF1	0.431289	1.34E-06
LARP1	-0.43518	1.05E-06	PAIP2B	-0.44454	5.76E-07
LHX8	-0.44417	5.90E-07	PAX9	-0.42377	2.14E-06
LINC00115	0.436236	9.83E-07	PDIA3P1	-0.43977	7.84E-07
LINC00667	0.447154	4.85E-07	PEX12	0.450037	4.01E-07
LOC100133331	0.537148	5.09E-10	PHLDB1	-0.43318	1.19E-06
LOC100190986	0.456057	2.68E-07	PICALM	-0.41806	3.02E-06
LOC105376064	-0.45383	3.11E-07	PICSAR	-0.49508	1.61E-08
LOC155060	0.428924	1.56E-06	PLEKHG4B	-0.44442	5.80E-07
LOC339803	-0.43273	1.23E-06	POLE2	-0.42566	1.90E-06
LRRC58	0.452839	3.33E-07	POLRMT	0.46302	1.66E-07
MALT1	0.442049	6.77E-07	PRM2	-0.47985	5.03E-08
MAN1B1-AS1	-0.44071	7.38E-07	PSEN2	-0.4222	2.35E-06
MAN2C1	0.509642	5.13E-09	PTMS	-0.45041	3.91E-07
MAP6	-0.42999	1.46E-06	RAC3	-0.44886	4.33E-07
MBTPS2	0.430662	1.40E-06	RETNLB	-0.42671	1.78E-06
MCTS1	0.464661	1.48E-07	REXO2	-0.45318	3.25E-07
MED1	0.463796	1.58E-07	RGR	0.443725	6.07E-07
MED12	0.467113	1.25E-07	RNASE13	-0.50999	4.99E-09
MED13L	0.465272	1.42E-07	RNF19A	0.444948	5.61E-07
MELTF-AS1	0.450022	4.01E-07	RORC	-0.43386	1.14E-06
METTL16	0.50737	6.15E-09	SCGB1C1	-0.462	1.79E-07
MEX3C	0.490471	2.28E-08	SEC16A	0.433199	1.19E-06
MFHAS1	0.504891	7.49E-09	SEPT10	-0.42131	2.48E-06
MICA	0.460144	2.03E-07	SHANK2-AS3	-0.43408	1.13E-06
MICAL1	0.500458	1.06E-08	SHOX	-0.45754	2.42E-07
MIR4435-2HG	-0.43848	8.52E-07	SIX5	-0.46458	1.49E-07
MIR497HG	0.436924	9.41E-07	SLC25A39	-0.49092	2.21E-08
MIS12	0.446931	4.92E-07	SLC25A5-AS1	0.455331	2.81E-07
MLLT10	0.506875	6.40E-09	SLC44A5	0.428986	1.55E-06
MNS1	0.449176	4.25E-07	SLC52A3	-0.43944	8.01E-07
MRPL39	0.429589	1.49E-06	SNAPC4	0.418683	2.91E-06
MRPL49	-0.43789	8.85E-07	SOX18	-0.44	7.73E-07
MSANTD2	0.459821	2.07E-07	SPINK2	0.427533	1.70E-06
MTRF1L	0.417645	3.09E-06	SPRR1A	-0.43193	1.29E-06
MUC20	0.47993	5.00E-08	SRC	0.460017	2.05E-07
MXD1	0.478273	5.64E-08	SSRP1	0.447862	4.63E-07
MXD4	0.459425	2.13E-07	STAMBP	-0.44861	4.41E-07
MYCNOS	-0.42493	1.99E-06	STK35	0.440607	7.43E-07
MYO5C	0.419267	2.81E-06	SUGP1	0.431965	1.29E-06
NCK2	0.418022	3.03E-06	TAAR8	-0.43308	1.20E-06
NCLN	0.455115	2.86E-07	TAS1R1	-0.45195	3.53E-07
NDUFA2	-0.47118	9.39E-08	TBC1D22B	0.432388	1.25E-06
NDUFV3	-0.48252	4.13E-08	TBC1D28	-0.43703	9.34E-07
NEIL2	-0.47934	5.22E-08	TCF15	-0.45994	2.06E-07
NELFCD	0.431165	1.35E-06	TCF20	0.461805	1.81E-07

NOL10	0.448784	4.36E-07
NOL11	0.472949	8.28E-08
NRF1	0.419248	2.81E-06
NSUN5P2	0.443421	6.19E-07
NTM	0.568902	2.69E-11
NTN5	0.464968	1.45E-07
NUP155	0.437951	8.81E-07
NUP43	0.439961	7.75E-07
NUTF2	-0.45814	2.33E-07
NXF1	0.429555	1.50E-06
NXPH3	0.437893	8.84E-07
ORC2	0.529586	9.81E-10
P3H2	-0.44504	5.57E-07
PER1	0.430178	1.44E-06
PHACTR4	0.514256	3.53E-09
PHGR1	-0.42058	2.59E-06
PIGP	-0.46724	1.24E-07
PILRA	0.461747	1.82E-07
PLEKHM1	0.447538	4.73E-07
PNISR	0.432376	1.25E-06
POGK	0.43684	9.46E-07
POLDIP3	0.495403	1.57E-08
POLG2	0.424899	1.99E-06
POLN	0.458387	2.29E-07
PPM1B	0.465975	1.35E-07
PPM1D	0.476616	6.36E-08
PPP1CB	0.463761	1.58E-07
PPP1R2	-0.43027	1.43E-06
PRKAB1	0.436421	9.71E-07
RAB39B	-0.43929	8.09E-07
RABL6	0.434868	1.07E-06
RAC2	-0.48635	3.11E-08
RALA	-0.4378	8.90E-07
RALGPS1	0.433076	1.20E-06
RAPSN	-0.46448	1.50E-07
RBM3	-0.43558	1.02E-06
REPS1	0.419552	2.76E-06
RESP18	-0.52659	1.27E-09
RHOT2	0.419763	2.72E-06
RING1	0.454097	3.06E-07
RNF115	0.426483	1.81E-06
RRN3P3	0.431692	1.31E-06
RRP8	0.470723	9.70E-08
RRS1	0.494961	1.62E-08
SAR1A	-0.42361	2.16E-06
SCPEP1	-0.42609	1.85E-06
SDF2L1	-0.44543	5.43E-07
SEC31B	0.446804	4.97E-07
SEC63	-0.48509	3.42E-08
SELENOO	0.445532	5.40E-07
SF3B1	0.446554	5.05E-07
SHISA4	-0.45068	3.84E-07
SHROOM3	0.420467	2.61E-06
SIRT7	0.459655	2.10E-07
SLC25A41	0.447112	4.87E-07
SLC29A4	-0.4226	2.30E-06
SMIM14	-0.51237	4.12E-09
SMYD4	0.462504	1.72E-07

TDRD10	-0.4212	2.50E-06
TEX44	-0.47614	6.58E-08
TFDP2	-0.43978	7.84E-07
TFF1	-0.44822	4.52E-07
TIMM29	0.527812	1.14E-09
TM9SF1	-0.47104	9.48E-08
TMA16	-0.42442	2.05E-06
TMEM182	-0.4492	4.24E-07
TMEM251	0.434722	1.08E-06
TMEM30B	-0.45908	2.18E-07
TMEM72	-0.4226	2.30E-06
TRPM5	-0.42223	2.35E-06
TTI1	0.459122	2.17E-07
TXNDC16	0.441445	7.04E-07
UBXN4	-0.45169	3.59E-07
ULBP1	-0.42808	1.64E-06
USP21	0.433484	1.17E-06
ZBTB7C	0.424896	1.99E-06
ZFP62	0.446835	4.96E-07
ZNF395	0.440684	7.39E-07
ZNF436-AS1	0.419556	2.76E-06
ZNF736	0.475902	6.70E-08
ZNF768	0.425092	1.97E-06

SNCG	-0.45716	2.49E-07
SON	0.426422	1.82E-06
SOX12	0.47163	9.09E-08
SPAG9	0.426279	1.83E-06
SPATA18	-0.46507	1.44E-07
SPSB3	0.428851	1.56E-06
SRRM3	0.428017	1.65E-06
STARD3NL	-0.44542	5.44E-07
SUPV3L1	0.455238	2.83E-07
TAGLN2	-0.44708	4.88E-07
TARBP2	0.459352	2.14E-07
TATDN3	-0.4874	2.88E-08
TIAL1	0.47439	7.47E-08
TM9SF3	-0.41876	2.89E-06
TMEM161B-AS1	0.44186	6.85E-07
TNFRSF11B	0.444536	5.76E-07
TP53I3	-0.44542	5.44E-07
TPRN	0.427663	1.68E-06
TRAF2	0.459998	2.05E-07
TRIM15	-0.41863	2.92E-06
TSHZ1	0.441945	6.82E-07
TTC17	0.464576	1.49E-07
TWISTNB	-0.47021	1.01E-07
TXNRD2	0.443152	6.30E-07
UBAC1	0.419406	2.78E-06
UBAC2	0.462469	1.73E-07
UBE2CP5	0.43047	1.41E-06
UBE2G2	0.439273	8.10E-07
UFM1	-0.46693	1.27E-07
ULK3	0.459698	2.09E-07
USP4	0.453205	3.25E-07
UTP11	-0.48016	4.91E-08
UTP14C	0.425157	1.96E-06
VAMP2	-0.42782	1.67E-06
VCP	-0.41918	2.82E-06
VN1R1	0.446343	5.12E-07
VPS13C	0.508889	5.45E-09
VTI1A	-0.46821	1.16E-07
WBP11	-0.42686	1.77E-06
WDFY3	0.483455	3.86E-08
WDR73	0.419248	2.81E-06
WDR81	0.582757	6.74E-12
XBP1	0.427521	1.70E-06
YPEL4	0.425199	1.96E-06
ZC3H18	0.423673	2.15E-06
ZFP82	0.433168	1.19E-06
ZFP91	0.439711	7.87E-07
ZGRF1	0.434368	1.11E-06
ZHX1	0.421716	2.42E-06
ZNF124	0.436644	9.58E-07
ZNF133	0.438604	8.45E-07
ZNF148	0.460759	1.94E-07
ZNF212	0.486485	3.08E-08
ZNF266	0.446362	5.11E-07
ZNF276	0.494634	1.66E-08
ZNF280D	0.510838	4.66E-09
ZNF302	0.426983	1.75E-06
ZNF32	0.424557	2.04E-06

ZNF384	0.449468	4.16E-07
ZNF563	0.431696	1.31E-06
ZNF564	0.420648	2.58E-06
ZNF576	0.436144	9.89E-07
ZNF579	-0.45599	2.69E-07
ZNF598	0.482575	4.12E-08
ZNF605	0.441595	6.97E-07
ZNF629	0.545271	2.47E-10
ZNF641	0.428125	1.63E-06
ZNF768	0.461501	1.85E-07
ZNF776	0.440934	7.28E-07
ZNF783	0.449975	4.03E-07
ZNF785	0.443936	5.99E-07
ZNF79	0.485758	3.25E-08
ZNF846	-0.43859	8.46E-07
ZNF862	0.433399	1.18E-06
ZNHIT1	-0.43809	8.73E-07
ZSCAN29	0.445924	5.26E-07
ZSCAN31	0.421928	2.39E-06
ZW10	0.425215	1.96E-06

ROI5

Gene	rho	p
AFAP1L1	0.464725	1.48E-07
FRMD8	0.428205	1.63E-06
LDLR	0.46145	1.85E-07
NPPC	0.473075	8.20E-08
NRM	0.436001	9.98E-07
SDR16C5	0.464283	1.52E-07
SYT6	-0.42359	2.16E-06

ROI6

Gene	rho	p
CMBL	-0.44801	4.59E-07
GDAP1	-0.42013	2.67E-06
SYVN1	0.474743	7.28E-08

ROI8

Gene	rho	p
C15orf40	-0.4282	1.63E-06
PTGR1	-0.41754	3.11E-06
RARS	-0.42025	2.65E-06
